# “Glyco-sulfo barcodes” regulate chemokine receptor function

**DOI:** 10.1007/s00018-023-04697-9

**Published:** 2023-02-02

**Authors:** Lisa Verhallen, Jarkko J. Lackman, Rikke Wendt, Martin Gustavsson, Zhang Yang, Yoshiki Narimatsu, Daniel M. Sørensen, Kato Mac Lafferty, Mieke Gouwy, Pedro E. Marques, Gertrud M. Hjortø, Mette M. Rosenkilde, Paul Proost, Christoffer K. Goth

**Affiliations:** 1 Laboratory for Molecular Pharmacology, Department of Biomedical Sciences, Faculty of Health and Medical Sciences, University of Copenhagen, Panum Building 185. Blegdamsvej 3B, 2200 Copenhagen, Denmark; 2 Laboratory of Molecular Immunology, Department of Microbiology, Immunology and Transplantation, KU Leuven, Louvain, Belgium; 3 Copenhagen Center for Glycomics, Department of Molecular and Cellular Medicine, Faculty of Health and Medical Sciences, University of Copenhagen, Copenhagen, Denmark

**Keywords:** Chemokine receptor, G protein-coupled receptor, O-glycosylation, Tyrosine sulfation

## Abstract

Chemokine ligands and receptors regulate the directional migration of leukocytes. Post-translational modifications of chemokine receptors including O-glycosylation and tyrosine sulfation have been reported to regulate ligand binding and resulting signaling. Through in silico analyses, we determined potential conserved O-glycosylation and sulfation sites on human and murine CC chemokine receptors. Glyco-engineered CHO cell lines were used to measure the impact of O-glycosylation on CC chemokine receptor CCR5, while mutation of tyrosine residues and treatment with sodium chlorate were performed to determine the effect of tyrosine sulfation. Changing the glycosylation or tyrosine sulfation on CCR5 reduced the receptor signaling by the more positively charged CCL5 and CCL8 more profoundly compared to the less charged CCL3. The loss of negatively charged sialic acids resulted only in a minor effect on CCL3-induced signal transduction. The enzymes GalNAc-T1 and GalNAc-T11 were shown to be involved in the process of chemokine receptor O-glycosylation. These results indicate that O-glycosylation and tyrosine sulfation are involved in the fine-tuning and recognition of chemokine interactions with CCR5 and the resulting signaling.

**Supplementary Information:**

The online version contains supplementary material available at 10.1007/s00018-023-04697-9.

## Introduction

Chemokine receptors belong to the large and diverse class A of the G protein-coupled receptors (GPCRs). Members of this class share a seven-transmembrane helices domain with a ligand-binding pocket and an intracellular C-terminus involved in G protein coupling and signal transduction. Traditionally, the interaction between chemokines and their receptors is described by the two-step/two-site model. During the first step, the core region of the chemokine binds to the N-terminal region and extracellular loops of the receptor. This chemokine recognition site 1 (CRS1) is important for the binding affinity. In the second step, the N-terminus of the chemokine is positioned in a way that it interacts with the extracellular loops and transmembrane domains of the receptor, called CRS2. This part will trigger a conformational change which leads to receptor activation [1, 2]. Although this model may need to be expanded or adjusted [3], the N-terminal region of chemokine receptors is indispensable for chemokine binding. Chemokine receptors have either reported or predicted sites of *N*-acetyl galactosamine (GalNAc)-type O-glycosylation in their N-termini [4]. In addition, they may be modified by tyrosine sulfation and a general co-localization of these two PTMs may be a common finding for chemotactic receptors [4]. These two post-translational modifications (PTMs) may act to fine-tune ligand affinity and downstream signaling. Activation of chemokine receptors can lead to a plethora of downstream effects, including cell proliferation and migration. The chemokine system appears as a highly promiscuous network as most chemokine receptors can bind multiple chemokines and vice versa. Its specificity and the regulation of the apparent promiscuity are still not completely understood. Differential expression of both chemokines and receptors in diverse tissues and cell types increases the intricacy of this system but may also provide a way to ensure proper immune responses [5, 6]. Some other mechanisms that can facilitate this specificity are for example the glycosaminoglycans (GAGs). GAGs are polysaccharides found on the cell surface and can bind to chemokines. This can influence chemokine/receptor interactions as some chemokines will become more rapidly immobilized to these GAGs and be better presented to leukocytes [6, 7]. Another example is the concept of biased signaling and the production of atypical chemokine receptors (ACKRs). GPCRs preferentially activate certain cellular signaling pathways which can be dependent on either ligands, receptors or cell type [5].

GalNAc-type or mucin-type O-glycosylation (hereafter called O-glycosylation) is initiated by the transfer of GalNAc to a serine or threonine residue (or in rare cases tyrosine). This initiation step is catalyzed by 20 different isoforms of polypeptide GalNAc-transferases (GalNAc-Ts) and the GalNAc is elongated further with the linkage of other monosaccharides by various glycosyltransferases to form diverse O-glycans [8]. Some O-glycosylation sites can be catalyzed by several different isoforms, whereas others are specifically controlled. Likewise, some GalNAc-T isoforms are widely expressed and have broad specificities, whereas others are more restricted [8]. The most common O-glycan is the core-1 or T structure consisting of a GalNAc with galactose in a β1,3-linkage, but there are several other core structures that can be further elongated and branched. The last step is usually the terminal capping by a single sialic acid, however, in rare cases glycans can carry polysialylation (PolySia), a homopolymer of α2,8-linked sialic acid, which can be extended with as many as several hundred units [9]. Polysialylation is well known for its role in neuronal development through its carrier protein neural cell adhesion molecule (NCAM). However, the function and distribution of PolySia are broader and include chemokine interactions with CCR7 [10] and other surface receptors [11, 12]. Importantly, the α2,8-sialyltransferases (ST8SIA), which synthesize PolySia are widely expressed in the immune system, with interesting dynamic expressions in different immune cells [13].

As we have previously reported [4], all CCRs are predicted to carry O-glycans in their N-terminus and a few have been experimentally verified. CCR5 carries in its N-terminal region up to four sialylated O-glycans which are important for CCL3 and CCL4 binding, whereas the little effect on HIV infection was observed [14]. Moreover, leukocytes carry distinct patterns of CCR7 sialylation, which contribute to receptor signaling and fine-tuning of chemotactic responses [15, 16]. CCR7 can be polysialylated which specifically affects the recognition of CCL21 but not CCL19 and consequently, dendritic cell trafficking [10]. The N-terminus of the cytomegalovirus chemokine receptor US28 is also O-glycosylated [17] and indirect evidence suggests that O-glycans differentially contribute to CC or CX3C chemokine binding [18]. The latest example involves GPR15, also a chemoattractant receptor, in which tyrosine sulfation improves ligand binding but O-glycosylation suppresses it [19].

Tyrosine sulfation takes place in the Golgi where the enzymes protein-tyrosine sulfotransferase 1 or 2 (TPST1/2) catalyze the transfer of a sulfate group from the adenosine 3’-phosphate 5’-phosphosulfate (PAPS) donor to the hydroxyl group of a tyrosine residue of the protein chain [20]. Tyrosine sulfation is heterogeneous and may show different occupancy in different cell lines or tissues [21–23]. Known tyrosine sulfation sites are present on membrane bound and secreted proteins involved in a broad range of functions, including hemostasis, regulation of the immune system and host–pathogen interactions [24]. Tyrosine sulfation on chemokine receptors boosts the affinity of chemokines through the charge interactions between the negative sulfates and positive regions of the chemokines [25]. Some studies show that combinations of tyrosine sulfation sites in chemokine receptor N-termini can differentially affect specific chemokines, suggesting that this acts as a fine-tuning mechanism of the chemokine system [25, 26]. Sulfation of the tyrosine residues of CCR5 plays a major role in promoting the interaction with CCL3 and CCL5 and is also important for HIV infection [14]. Evidence suggests that specific sulfation sites may differentially affect CCL3 and CCL5 binding [14] and that sulfation on CCR5 is heterogenous with a potential effect on its binding properties to chemokines [23]. Several other chemoattractant receptors including receptors for C5a and C3a, atypical chemokine receptor 2 (ACKR2), US28 and sphingosine-1-phosphate receptor 1 (S1PR1) also carry tyrosine sulfation sites in their N-termini with implications for ligand binding and signaling [14, 18, 27–29].

Despite the growing body of evidence for a general and widespread occurrence of these patterns or “GlycoSulfo barcodes” in chemotactic receptors, there is still limited research on the subject. One reason has been the paucity of technologies and methods to study the functional impact of O-glycosylation. However, recent progress in genetic engineering has significantly expanded the possibilities. Here, we employ isogenic glycoengineered cell lines, mutations and specific inhibitors to dissect the role of O-glycosylation and the interplay with tyrosine sulfation on chemokine receptor function.

## Material and methods

### Cell cultures

CHO cells were grown in suspension under serum-free conditions in CHO medium consisting of a 1:1 ratio of BalanCD^®^ CHO GROWTH A medium (Irvine Scientific, Santa Ana, CA, United States) and EX-CELL^®^ CD CHO Fusion medium (SAFC, St Louis, MO, United States) with 2% GlutaMAX (Gibco, Carlsbad CA, United States) and 1% penicillin and streptomycin at 37 °C and 10% CO_2_. The THP-1 cells (ATCC, TIB-202, Manassas, VA) were grown in suspension in RPMI with GlutaMAX (Gibco) 10% Fetal Bovine Serum (FBS) (Sigma-Aldrich, Saint Louis, MI, United States) and NaHCO_3_ (Gibco) at 37 °C and 5% CO_2_. HEK293 were grown in DMEM (Sigma-Aldrich) supplemented with 10% heat-inactivated fetal bovine serum (Sigma-Aldrich) and 2 mM GlutaMAX (Gibco).

### pERK assay

THP-1 cells were incubated overnight in a serum-free starvation medium (RPMI medium without FCS) (Gibco). The following day, THP-1 cells were suspended at a concentration of 1 × 10^6^ cells/ml in serum-free starvation media with 32 mU/ml or 16 mU/ml neuraminidase (Sigma) diluted in PBS or a similar amount of vehicle control and incubated for 1 h at 37 °C. Afterwards, the cells were resuspended at 8 × 10^6^ cells/ml in serum-free starvation medium supplemented with 0,5% BSA (Sigma) and stimulated with 30 ng/ml CCL5 (diluted in starvation medium + 0.5% BSA) (Peprotech) or starvation medium. After 2 min, signal transduction was stopped by adding ice-cold PBS. The cells were centrifuged and cell lysis (90 µl lysis buffer/sample) was performed in PBS containing 1 mM EDTA, 0.5% Triton X-100, 5 mM NaF, 6 M urea, protease inhibitor cocktail for mammalian tissues and phosphatase inhibitor cocktails 1 and 2 (Sigma). The lysate was incubated for 15 min on ice and the supernatant was collected after centrifugation (8 min, 400*g*). The protein concentration in the supernatant was determined by the bicinchoninic acid (BCA) protein assay (Pierce, Rockford, IL, USA). The amount of phosphorylated ERK1/2 in the supernatant was determined using a duoset ELISA for phospho-ERK1 and phospho-ERK2 (R&D systems). The ratio of phospho-ERK1/2 to total protein content was calculated for cell lysates. The results are expressed relative to the control group.

### Flow cytometry staining

For live-dead staining, the THP-1 cells were treated with neuraminidase as described for the pERK assay. Afterwards the THP-1 cells were treated with Fixable Viability Stain 620 (BD Biosciences, Franklin Lakes, NJ, US) for 15 min at room temperature. Subsequently, the cells were washed with flow cytometry buffer (PBS + 2% FCS + 2 mM EDTA; Sigma). Separately, THP-1 cells were stained with CCR1-AF647 (557,914, BD Pharmingen) and CCR5-BV421 (5,562,576, BD Horizon) or biotinylated Peanut agglutinin PNA (B-1075–5, Vectorlabs, CA, US) and streptavidin-PE (554,061, BD Pharmingen). Results were analyzed using a BD LSRFortessa™ X-20 flow cytometer (BD Biosciences) equipped with DIVA software (BD Biosciences). FlowJo software (BD Biosciences) was used for analysis.

### Cell lines

The glycoengineered CHO/HEK cells were developed using CRISPR/Cas9 technology to knock-out or knock-in individual glycogenes as previously described at the Copenhagen Center for Glycomics. Cell lines were validated before use by genomic sequencing. The wild type (WT) CHO produces core-1 O-glycans and has five GalNAc-Ts transcribely expressed. The SimpleCell (SC) cells have the *COSMC* gene knocked out, making them unable to elongate the initial GalNAc. The 5XKO cells have all *Galnt* genes knocked out which are *Galnt1, Galnt2, Galnt7, Galnt10* and *Galnt11*. The CHO 3XKO are KO of *Galnt1, Galnt2* and *Galnt11*. The T3KI cells has human *GALNT3* introduced. The ∆Sia cell lines have the *St3gal1and 2* gene knocked out leading to a lack of sialylation of O-glycans. The PolySia cell line has the polysialyltransferase human *ST8SIA4* knocked in, which facilitates polysialylation. Individual Galnt knock-outs of *Galnt1, Galnt2* or *Galnt11* were also used. For HEK293 cells, WT, SC (KO *COSMC*) and 3XKO (KO *GALNT1/2/3*) cell lines were used.

### Transient transfection of CHO and HEK293 cells

The transfection mix was made consisting of 200 µl serum-free medium (Opti-SFM, Gibco) with 0.2 µg receptor and 0.8 µg CAMYEL sensor (cAMP sensor using YFP-Epac-RLuc) for BRET experiments or 1 µg receptor for western blotting for each transfection of 2 × 10^6^ cells. At the end, 4 µl of FectoPro (Polyplus, Illkirch, France) was added to each transfection and the mixture was kept at room temperature for 15 min. Cells were resuspended in 500 µl serum-free medium (Opti-SFM, Gibco) with 1% GlutaMAX (Gibco) per 2 × 10^6^ cells. Next, they were transferred to a 6-well plate at 2 × 10^6^ cells/well and incubated at 37 °C. Afterwards, 200 µl of the mixture was added and the cells were incubated for 3 h at 37 °C and 10% CO_2_. After incubation, 2 µl of FectoBooster (Polyplus) was added together with 2.5 ml of CHO media without penicillin and streptomycin to each cell. For inhibition of tyrosine sulfation, 100 mM sodium chlorate (NaClO_3_) (Sigma-Aldrich) was added to the CHO media without penicillin and streptomycin. The cells were incubated overnight at 37 °C and 10% CO_2_. HEK293 cells were transfected using Lipofectamine2000 transfection reagent (Thermo Fisher Scientific), according to the manufacturer’s instructions. The culture medium was changed to Opti-MEM (Gibco) before transfection, and changed back to the appropriate medium 3 h after transfection.

### Western blotting

CHO cell lysates were made by washing in PBS and resuspending the transfectants in 250 µl lysis buffer consisting of 10 × RIPA buffer (Millipore, Merck, Burlington, MA, United States), ultrapure water and 7 × protease inhibitor (Roche, Basel, Switzerland). For gel electrophoresis, 30 µl of CHO cell lysate were mixed with 11 µl of 4 × sample buffer (Invitrogen, Carlsbad, CA, United States) and 4 µl of 3 M dithiothreitol (DTT) (Sigma-Aldrich). The samples were loaded on a 4–15% gradient gel and an electrophoresis buffer of 10 × tris–glycine-SDS (BIO-RAD, Hercules, CA, United States) and ultrapure water in a 1:9 ratio. Following the electrophoresis, the transfer was performed according to the Trans-Blot Turbo RTA transfer kit (BIO-RAD). After the transfer on an LV PVDF membrane, blocking was performed in Intercept™ blocking buffer (LI-COR, Lincoln, NE, United States) for 30 min. The antibody ANTI-FLAG^®^ M1, f3040 (Sigma-Aldrich) (1:400) was added to bind overnight. The following day the membrane was washed and a secondary antibody IRDye 800CW, goat anti-mouse (LI-COR) at 1:5000 was added. Alternatively, HRP-conjugated ANTI-FLAG M2 (Sigma-Aldrich) (1:30 000) was used, and the chemiluminescence was analyzed after incubation of the membrane with WesternSure PREMIUM chemiluminescent substrate (LI-COR), according to manufacturer’s instructions. For lectin-blotting detection of GalNAc-modified proteins, membranes were stained with biotinylated Vicia Villosa lectin (Vector Laboratories) (1:1000), followed by HRP-conjugated streptavidin (ThermoFisher Scientific) (1:2000). After the indicated staining’s, the membrane was analyzed by the Odyssey Fc Imaging System (LI-COR).

### Immunoprecipitation

An ANTI-FLAG M2 Affinity Gel (Sigma) was used for immunoprecipitation of FLAG-tagged receptors. The agarose was equilibrated twice with 0.1 M glycine–HCl, pH 3.5 and then three times with 50 mM Tris, pH 7.4. Finally, the agarose was equilibrated with lysis buffer containing 0,1% BSA and incubated in a rotor for 1 h at 4 °C. The agarose was spun down at 1000 g and the lysis buffer was removed. Cell lysates (400 µl) were then added and the samples were placed on a rotor overnight at 4 °C. The next day, the samples were spun down and the agarose washed 5 times with lysis buffer. Finally, immunoprecipitated samples were eluted by incubating in sample buffer for 15 min at room temperature followed by 5 min at 95 °C. Alternatively, FLAG-tagged receptors were eluted by 3xFLAG peptide by incubating 10 min on ice and collecting the eluate.

### Bioluminescence resonance energy transfer (BRET) cAMP assays

Twenty-four hours after transfection, the CHO cells were washed in PBS and afterwards resuspended in PBS + 1% glucose. Eighty µl of 4 × 10^6^ cells/ml were seeded in a 96-well plate (~100,000 cells/well). Five µM coelenterazine (NanoLight Technology, Pinetop, AZ, United States) was added to the cells. After 10 min, 5 µl of varying ligand concentrations was added to each well. After 15 min coelenterazine and 1 µM forskolin (Sigma-Aldrich) were added. The plates were kept in the dark all the time. The emission signals from *Renilla* luciferase (RLuc) and yellow fluorescent protein (YFP) were measured with a 2104 EnVision Multilabel plate reader (PerkinElmer, Waltham, MA, United States). The BRET signal is the ratio of the detected YFP (acceptor emission) at 525 nm divided by the RLuc at 475 nm (donor emission) [30].

### In vitro glycosylation and MALDI-TOF

Recombinant glycosyltransferases were expressed as soluble secreted truncated proteins in insect cells [31]. In vitro activity assays for GalNAc-T glycosylation of the CCR5 peptide (Synpeptide, Shanghai, China) were performed in 25 μL buffer (25 mM cacodylic acid sodium, pH 7.4, 10 mM MnCl_2_, 0.25% Triton X-100), with 2 mM UDP-GalNAc (Sigma), 10 μg of acceptor peptide and 0.1 μg of purified enzyme incubated at 37 °C. As a control for enzyme activity, a MUC1-60 mer peptide with sequence VTSAPDTRPAPGSTAPPAHGVTSAPDTRP APGSTAPPAHGVTSAPDTRPAPGSTAPPAHG (Synpeptide, Shanghai, China) was used. Reactions were monitored with Matrix-Assisted Laser Desorption/Ionization-Time of Flight (MALDI-TOF) mass spectrometry at given timepoints by removing 1 µl of reaction and mixing with 50 µl of 0,1% TFA in water. This was then mixed 1:1 with a solution of 2,5-dihydroxybenzoic acid on a steel target plate and analyzed on a Bruker Autoflex instrument (Bruker Daltonik GmbH, Bremen, Germany) in linear positive mode.

### Modelling of chemokines and receptors

The electrostatic surfaces of CCL3 (PDB ID: 5COR), CCL5 (5COY) and CCL8 (1ESR) were calculated using the APBS electrostatics plugin in PyMol. Chemokines were aligned to the crystal structure of the CCR5:CCL3 complex (7F1T) or to the NMR structure of CCL5 bound to a peptide corresponding to the N-terminus of CCR5 (6FGP) to visualize the position of potentially sulfated or glycosylated residues.

## Results

### A general pattern of O-glycosylation and tyrosine sulfation in C–C chemokine receptors

**Fig. 1:**
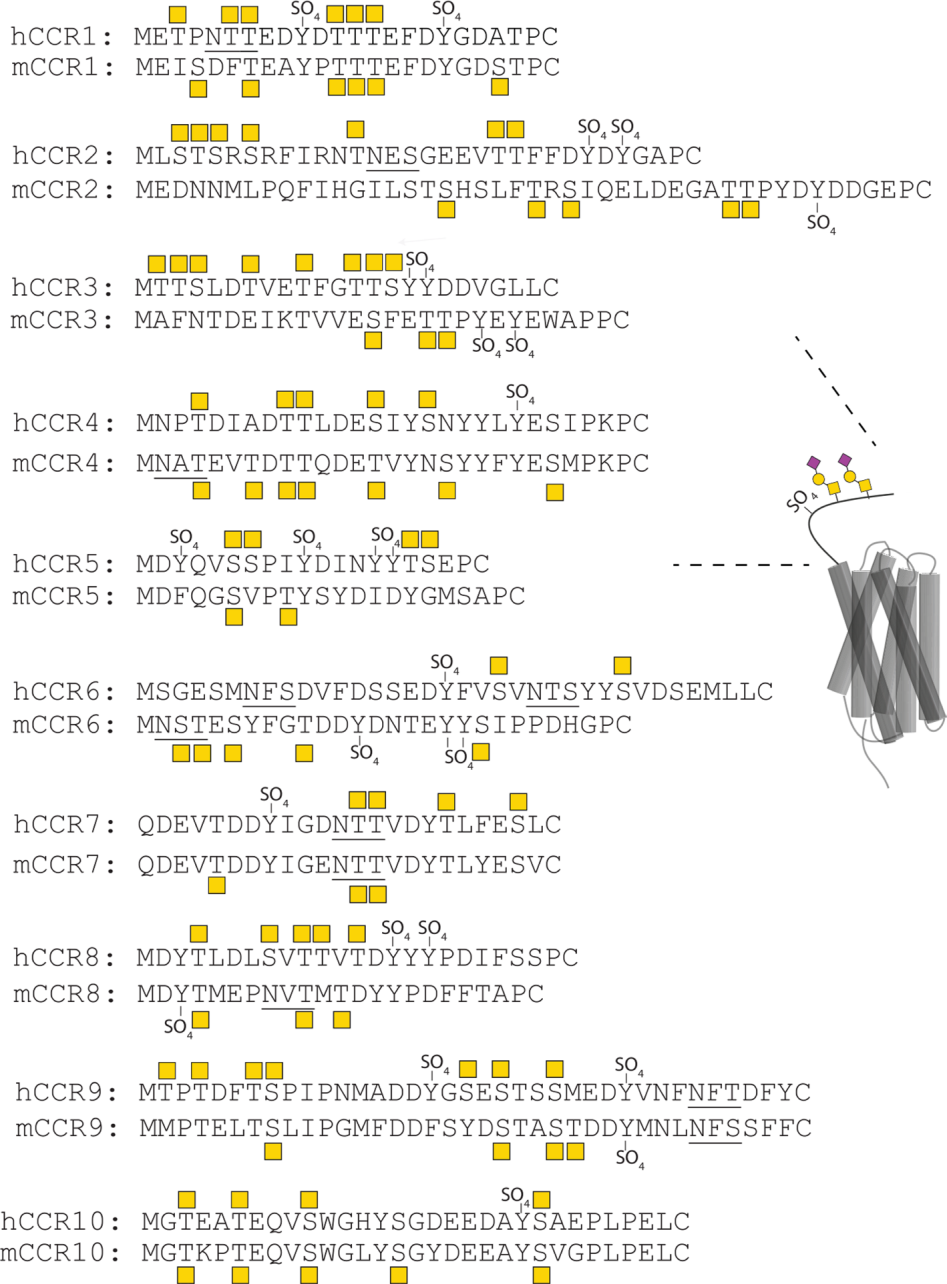
In silico analysis reveals a general and conserved “Glyco-Sulfo barcode” in C–C Chemokine Receptor N-termini. N-terminal sequences from human (h) and mouse (m) CCRs are aligned and predicted potential O-glycosylation and tyrosine sulfation sites are indicated on the human sequences with a yellow square or SO_4_, respectively. Consensus sequences for N-glycosylation are underlined. Potential N-glycosylation motifs are underlined

To map the presence of possible O-glycosylation and tyrosine sulfation sites, we analyzed the sequences corresponding to the N-terminal domains of all CCRs with the NetOGlyc 4.0 prediction algorithm [32] for O-glycosylation and the sulfinator tool for tyrosine sulfation sites [33]. We also mined the databases glycodomain viewer [32] for identified O-glycosylation and GPS-TSP for tyrosine sulfation sites [34]. As shown in Fig. 1, all CCRs have potential O-glycosylation as well as tyrosine sulfation sites. Consensus sequences for N-glycosylation are present in 5 out of 10 human CCRs and conserved in mouse CCR4, CCR7 and CCR9. The potential presence of O-glycosylation and sulfation sites furthermore seems to be widely conserved between human and murine sequences. Although tyrosine sulfation generally enhances chemokine affinity, it may lead also to more distinct effects as reported for CCR3 in which differentially sulfated peptides display selective binding of chemokines [35].

### Neuraminidase inhibits CCL5-mediated ERK phosphorylation in THP-1 cells

CCR1, CCR3 and CCR5 have multiple shared chemokine ligands, creating the need for a regulatory mechanism controlling ligand binding. Both CCR1 and CCR5 are expressed in monocytes and dendritic cells. While CCR3 is mainly expressed in eosinophils. Also all of them can be found on T cells [36]. We, therefore, decided to focus on these three receptors in the present study.

**Fig. 2:**
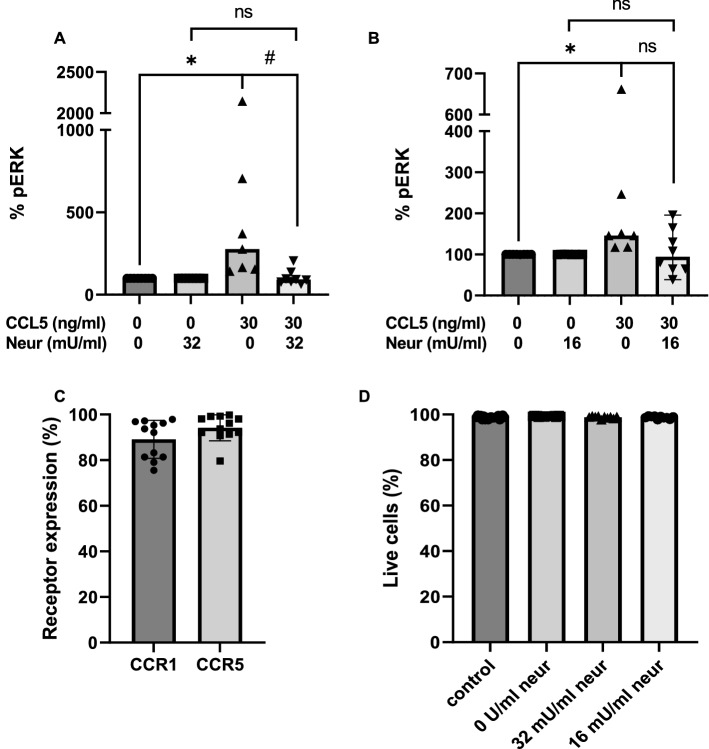
Neuraminidase treatment inhibits phosphorylation of ERK1/2. THP-1 cells were stimulated with **A** 32 mU/ml or **B** 16 mU/ml neuraminidase dissolved in PBS. A similar amount of PBS was added to the control group. The cells were incubated for 2 min in a medium with or without CCL5. The amount of phosphorylated ERK1/2 (pERK) was determined by ELISA. The median values were determined for 7 to 8 experiments. 100% corresponds to the amount of pERK in medium-treated cells. Phosphorylation of ERK1/2 induced by CCL5 was compared and statistical significance was evaluated by the sign test; comparison to the medium-treated cells (*p < 0,05); comparison between groups with or without neuraminidase (#*p* < 0,05); ns = not significant. **C** The mean ± SEM percentage CCR1 and CCR5 receptor positive THP-1 cells of 12 experiments determined by flow cytometry. **D** The mean values ± SEM of 10 experiments of live THP-1 cells were determined by flow cytometry with FVS620 staining and the percentage of live cells are shown for each group

To confirm the results obtained in silico*,* we initially probed the functional effect of endogenously expressed CCRs after removing the terminal sialic acids on glycan chains. Neuraminidase treatment has previously been shown to affect chemokine binding to transiently expressed CCR5 in CHO cells [14]. We chose to test the role of sialylation in the human monocyte-like cell line THP-1, which expresses CCR1 and CCR5 endogenously (Fig. S1). Cells were treated with neuraminidase, resulting in cleavage of terminal sialic acids from the cell surface. Subsequently, signaling was measured using an ERK1/2 phosphorylation (pERK) assay after incubation of the THP-1 cells with CCL5 (a chemokine ligand for both CCR1 and CCR5) for 2 min. The amount of ERK1/2 phosphorylation was significantly lower in the cells treated with 0,032 U/ml neuraminidase, whereas the observed decrease was not significant for the lower concentration of 0016 U/ml (Fig. 2). To verify that the effect of neuraminidase was not due to toxicity, we performed a live/dead stain of cells from the different treatments, which showed that there was no effect on the viability of the cells after the neuraminidase treatment (Fig. S2). This experiment verified that cell surface sialic acids are important for signaling of endogenous CCRs, possibly due to O-glycosylation in their N-termini, since CCR5 has no N-glycosylation motifs However, because neuraminidase may cleave multiple available sialic acids from the cell surface, more experiments are needed to confirm this hypothesis.

### CCR1 and 5 are O-glycosylated in CHO and HEK293 cells

Previous studies that explored the role of glycosylation on CCRs have been limited to the use of enzymatic treatments (e.g., with neuraminidase) or a mutational approach changing individual amino acids in transfected constructs. These tools are valuable and have provided important insights, nevertheless, they also have shortcomings. Changing individual amino acids when mutating glycosylation acceptor sites may have indirect effects such as affecting other nearby PTM sites or general properties of the carrier proteins. However, recent progress in genetic engineering capabilities has presented other avenues and we and others have developed broad platforms including knock-out and knock-in of glycosyl transferases that allow for new detailed dissections of the functional effect of glycans [37, 38].

**Fig. 3:**
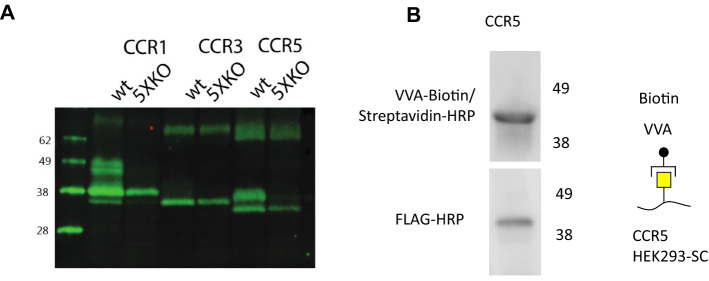
Western blot analysis of CCR transiently transfected CHO and HEK293 cells showing O-glycans. A Cells were transfected with a FLAG-tagged CCR1, CCR3 or CCR5 construct, harvested and analyzed by western blot 24 h later. The decrease in size between WT and 5XKO (CHO cell lacking 5 different GalNAc-Ts) shows that CCR1 and CCR5 are glycosylated in these CHO cells. B CCR5 was transiently expressed in HEK293 SCs and cellular lysates were prepared 24 h after transfection. Flag-tagged CCR5 was immunoprecipitated with FLAG M2 antibody and analyzed by WB and lectin blotting, using FLAG M2-HRP or biotinylated-VVA followed by streptavidin-HRP, respectively

Cells and tissues often show diverse and distinct expressions of GalNAc-Ts [8, 39], the enzymes responsible for initiating O-glycosylation. Because no information is available on GalNAc-T specificity for CCR sequences, it is not known which cell lines will O-glycosylate CCRs and to which degree. To probe this rationally, CCR1, CCR3 and CCR5 were transfected into the series of the GalNAc-T knock-out isogenic cell library as well as cosmic knock-out which led to presenting only truncated O-glycan, so-called ‘Simple-Cell (SC)’ in CHO and HEK293 cells (Fig. 3 and Fig. S2). We first transfected HEK WT, SC and a knock-out of 3 different commonly expressed GalNAc-Ts (GALNT1, 2 and 3) “3XKO”. As SC only present truncated short O-glycan, we clearly observed a mobility shift in the western blot (Fig. S2A) suggesting that all CCRs undergo O-glycosylation in HEK293 cells. We can also observe such mobility shift in 3XKO for CCR1, 2 and 3 but no mobility shift for CCR5 between WT and the 3XKO, which suggests that some of the remaining GalNAc-Ts are responsible for glycosylating CCR5 (Fig. S2A). Next, we used two different CHO cells, WT and “5XKO”, a clone that is a knock-out for 5 different GalNAc-Ts (GALNT1/2/7/19/11) and lost the O-glycosylation capabilities. The significant change in band mobility between CHO WT and 5XKO (Fig. 3) revealed that CCR1 and CCR5 carry O-glycosylations in CHO cell lines, whereas CCR3 (Fig. 3A) and CCR2 (Fig. S2B) do not. The signal of CCR2 was low and was therefore immunoprecipitated and analyzed separately. Consequently, we selected the CHO cells for downstream analysis as this allowed for more precise dissection of important GalNAc-Ts. Previously, it was shown that CCR5 contains sialylated GalNAc-type O-glycan [14]. To confirm that chemokine receptors are directly modified by GalNAc-type *O*-glycans in our expression systems, CCR5 was expressed in HEK293 SC to produce truncated *O*-glycans. The glycosylated CCR5 was then purified from cellular lysates and identified thereafter with biotinylated *Vicia Villosa* lectin (VVA) that recognizes α- or β-linked terminal GalNAc residues, especially single GalNAc residues linked to Ser/Thr in a polypeptide, as produced in HEK SCs (Fig. S2A). We have used the VVA lectin before to identify GalNAc-type *O*-glycans and it was established that it does not bind elongated *O*-glycans produced in our HEK293 WT cells (Fig. 3B, Fig S2C) [40]. Furthermore, CCR5 is not modified by *N*-glycans, excluding the possibility of other sources of GalNAc-residues in CCR5.

### O-glycosylation fine-tunes Gα_i_-signaling through CCR1 and CCR5

Next, the effect of O-glycosylation (both presence and glycan structure) on signaling by CCR5 and CCR1 was investigated by Bioluminescence Resonance Energy Transfer (BRET)-based signaling assays in cell lines with different glycosylation capacities. In the BRET assay, the elevation of intracellular cAMP levels leads to closer proximity between bioluminescent and fluorescent protein tags in the cytoplasm, allowing for energy transfer to happen. As chemokine receptors signal through the Gα_i_ protein, a reduction in cAMP artificially induced by forskolin can be measured after induction of signaling. During BRET, RLuc will emit blue light at 475 nm that overlaps with the excitation spectrum of YFP, creating an emission at 525 nm. These acceptor/donor ratios are measured to calculate the ΔBRET ratio, [30].

**Fig. 4:**
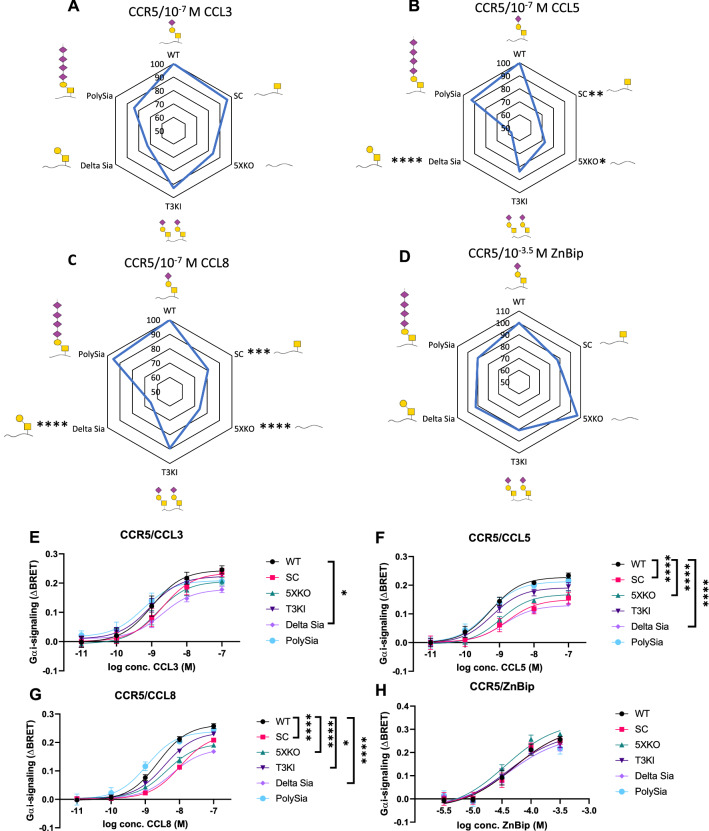
Analysis of CCR5-mediated signaling in CHO cell lines producing distinct O-glycoforms. CHO cell lines were transfected with CCR5 and stimulated with 100 nM **A** CCL3, **B** CCL5, **C** CCL8 or **D** 100 µM ZnBip. The figures show the radar plot presentation of the mean ΔBRET obtained in mutant CHO cell lines compared to the mean obtained in CHO WT cell line (normalized to 100 percent) in percentages of 3 (5xKO ZnBip = 2) independent experiments, performed in duplicate, 40 min after addition of the ligand. Statistically significant differences (*p* < 0.05) in comparison to WT for 10^–7^ M chemokine are indicated with an *. Glycans are shown as GalNAc = yellow square, Galactose = yellow circle and Sialic acid purple diamond. **E**–**H** Cells were stimulated with **E** CCL3, **F** CCL5, **G** CCL8 or **H** ZnBip. The results show the mean ΔBRET + standard error of the mean (SEM) of 3 (5xKO ZnBip = 2) independent experiments performed in duplicate 40 min after the addition of the ligand Statistically significant differences by a two-way ANOVA test and a multiple comparison Tukey test between WT and another cell line are highlighted for the whole curve with stars: ****p-value < 0.0001, ***p-value ≤ 0.001 and *p-value ≤ 0.05. Legend of symbols: Yellow square: GalNAc, yellow circle: galactose, purple triangle: sialic acid

The signaling properties in six different cell lines were compared; with some having reduced O-glycosylation capacities, due to the knock-out of one or more glycosyltransferases (SC, 5XKO and Delta Sia) and others with increased capacities due to a knock-in of glycosyltransferases (PolySia and T3KI) (Fig. 4). Activation of CCR5 by CCL3 was not significantly affected with the removal of glycosylation capacities. Especially with the SC cell line no difference was observed. Increasing the glycosylation capacities by a knock-in of GALNT3 (T3KI) or a knock-in of ST8SIA4 (PolySia) that increases the amount of sialic acids, also does not impact CCL3-induced Gα_i_-signaling significantly. Overall, CCL3-dependent signaling is affected marginally by CCR5 receptor glycosylation (Fig. 4A). However, different observations can be made for CCL5 or CCL8-induced Gα_i_-signaling (Fig. 4B, C). Removal of glycosylation by removing only the sialic acid (Delta Sia) or the whole glycan (5XKO) reduces the signaling noticeably indicating the importance of the terminal sialic acid in CCR5 signaling activated by CCL5 and CCL8. On the other hand and similarly as with CCL3, increasing the glycosylation capacities (T3KI and PolySia) does not affect signaling by these chemokines. Also, modification of the O-glycosylation pattern on CCR1 did not affect CCL3 signaling but had a major impact on CCL5-dependent Gα_i_-signaling (Fig. S4). In general, we observed reduced Gα_i_-signaling by CCR1 and CCR5 in cell lines with truncated O-glycans when stimulated with CCL5 or CCL8 and to a lesser extent by CCL3 (Fig. 4, Fig. S3–5, Table S1, 2).

Globally changing O-glycosylation may have a broad impact on receptor trafficking, localization, stability and receptor interactions. To confirm that the obtained results were due to differences in O-glycosylation, a small molecule metal ion chelator was used as a positive control, bipyridine complexed with Zn^2+^ (ZnBip) (Fig. 4D). ZnBip is a CCR5 agonist which interacts directly with the aromatic residues of the transmembrane domains in the CCR5 binding pocket [41]. Unlike, the chemokines, ZnBip, therefore, induces signaling independently of the receptor N-terminus making this compound an important control for indirect effects and general differences between cell lines (Thiele et al. 2011). The potency and efficacy of the Gα_i_-signaling after the addition of ZnBip was similar for all cell lines, verifying that the differences observed after chemokine addition are the consequence of the change in O-glycosylation (Fig. 4D). However, it is possible that cellular capacity for glycosylation may have contributed to the observed effect.

### The impact of O-glycosylation is affected by tyrosine sulfation status

**Fig. 5:**
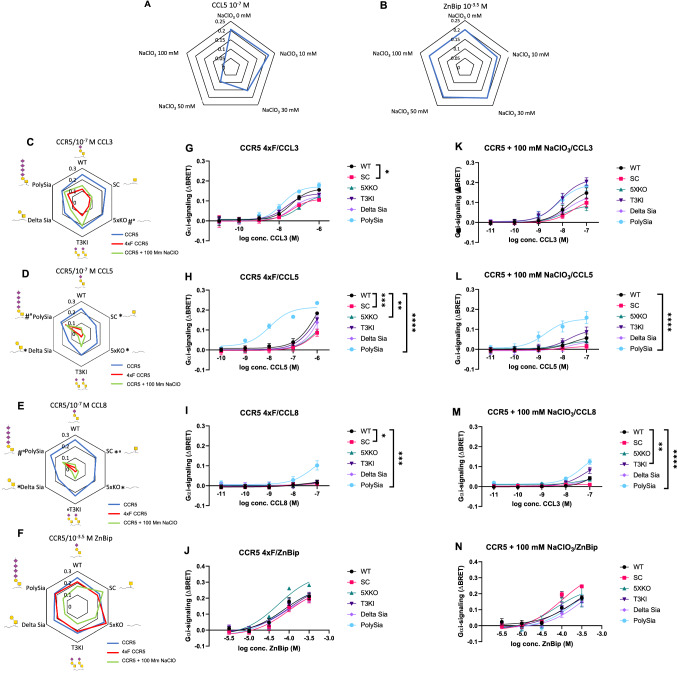
Analysis of CCR5-mediated signaling in CHO cell lines with changed tyrosine sulfation status. **A**, **B** Analysis of CHO cell lines transiently transfected with CCR5 and grown for 24 h in CHO medium with various concentrations of NaClO_3_. Cells were stimulated with 10^–7^ M CCL5 or 10^–3.5^ M ZnBip. The radar plots show the mean ΔBRET of, respectively, 3 (5xKO ZnBip = 1) independent experiment(s), performed in duplicate, 40 min after addition of the ligand. **C**–**F** Analysis of CHO cell lines transiently transfected with CCR5 (blue line), CCR5 4xF (red line) or CCR5 and grown for 24 h in CHO medium with 100 mM of NaClO_3_ (green line). Cells were stimulated with 100 nM CCL3, CCL5, CCL8 or 100 µM ZnBip. The radar plots show the mean ΔBRET obtained in mutant CHO cell lines of 3 independent experiments, performed in duplicate 40 min after the addition of the ligand. Statistically significant differences (*p* < 0.05) in comparison to WT for 10^–7^ M chemokine are indicated with an * for CCR5, # for 4xF CCR5 compared to CCR5 and ° for treatment with or without 100 mM NaClO_3_. **G**–**N** Cells were stimulated with **G**, **K** CCL3, **H**, **L** CCL5, **I**, **M** CCL8 or **J**, **N** ZnBip. The results show the mean ΔBRET ± standard error of the mean (SEM) of 3 (5xKO ZnBip = 1) independent experiments performed in duplicate, 40 min after the addition of the ligand. Statistically significant differences by a two-way ANOVA test and a multiple comparison Tukey test between WT and another cell line are highlighted for the whole curve with stars: ****p-value < 0.0001, ***p-value ≤ 0.001, **p-value ≤ 0.01 and *p-value ≤ 0.05. Legend of symbols: Yellow square: GalNAc, yellow circle: galactose, purple triangle: sialic acid

As shown in Fig. 1, tyrosine sulfation is potentially just as conserved as O-glycosylation in the CCR N-termini and both PTMs are suggested to play a role in the fine-tuning of chemokine signaling [42]. To investigate this in our setting, BRET experiments were performed where both O-glycosylation and tyrosine sulfation were modified for CCR5. To control tyrosine sulfation, all four tyrosine residues were mutated in the N-termini to phenylalanine residues, creating the CCR5 4xF mutant. In parallel, wild-type cells were cultured in 100 mM sodium chlorate (NaClO_3_) for 24 h and stimulated with CCL5 or ZnBip (Fig. 5A, B). This was the highest concentration that was not toxic and had a clear effect on Gα_i_-signaling. NaClO_3_ shuts off the sulfation of the whole cell by inhibiting ATP sulfurylase, the first enzyme in the 3ʹ-phosphoadenosine 5ʹ-phosphosulfate (PAPS) synthesis [43]. Consequently, NaClO_3_ removes sulfation in general and thus is much broader in its effect compared to the mutation of individual residues, making the two approaches complementary.

**Fig. 6:**
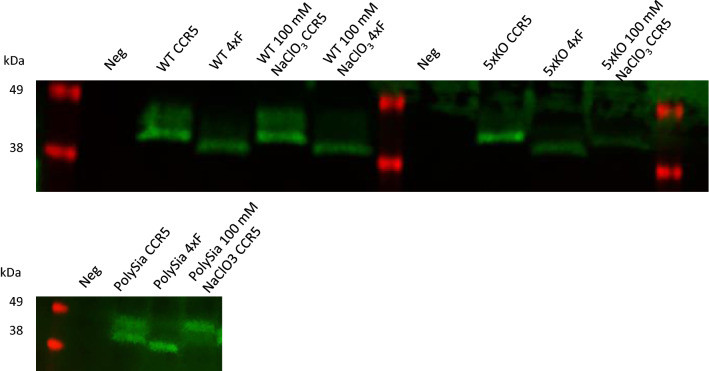
Differences in glycosyation are observed depending on the method of removal of tyrosine sulfation. CHO cells were transfected with FLAG-tagged CCR5, 4xF CCR5 or CCR5 and treated with 100 mM NaClO3, harvested and analyzed by western blot. It was revealed that the 4xF mutant in all cell lines apparently not only removes tyrosine sulfation but also O-glycosylation. The cells treated with 100 mM NaClO3 still have O-glycosylation. A downshift was detected in the cell lines transfected with the 4xF sequence. This is likely due to alterations in SDS binding which has been reported before in studies with tyrosine to phenylalanine mutations in GPCRs (13). (Full WB can be found in supplementary Figure. S4.)

As expected, we found that after the removal of tyrosine sulfation, the Gα_i_-signaling was significantly reduced both by mutation and NaClO_3_ treatment (Fig. 5C–F), Fig. S6, Table S3, 4). Although this effect was most pronounced for CCL5 and CCL8, CCL3 signaling was also significantly reduced. Surprisingly, we found that the ΔBRET value obtained with the “PolySia” cell line was similar when stimulated with 100 nM CCL5 and a similar pattern was found for CCL8, although with CCL8 significantly less signaling was observed compared to the “normal” sulfation status (Fig. 5D, E). For CCL3, no compensatory effect for the removal of sulfation was observed by polysialylation. We also observed retained signaling in the T3KI cells which expressed one additional GalNAc-T (GalNAc-T3) compared to the other cell lines. Especially the ΔBRET value obtained after 100 nM CCL3-induced signaling with or without 100 mM NaClO_3_ is not significantly different. (Fig. 5C). ZnBip was used to verify whether the results obtained were caused by the change in O-glycosylation and tyrosine sulfation (Fig. 5F). The ΔBRET values, after the addition of ZnBip was similar in all cell lines, confirming that the different ΔBRET values are driven by changes in the receptor N-termini. To investigate if inhibition of tyrosine sulfation affects O-glycosylation, western blots of cells transfected with the WT and 4xF mutant constructs from non-treated and NaClO_3_ treated cells were performed (Fig. 6). The 4xF showed a downwards shift due to the introduction of phenylalanine, (and effects previously reported [27]), whereas no difference was observed for NaClO_3_ treated cells.

### Knock-out of GalNAc-T1 reduces signaling induced on CCR5

**Fig. 7:**
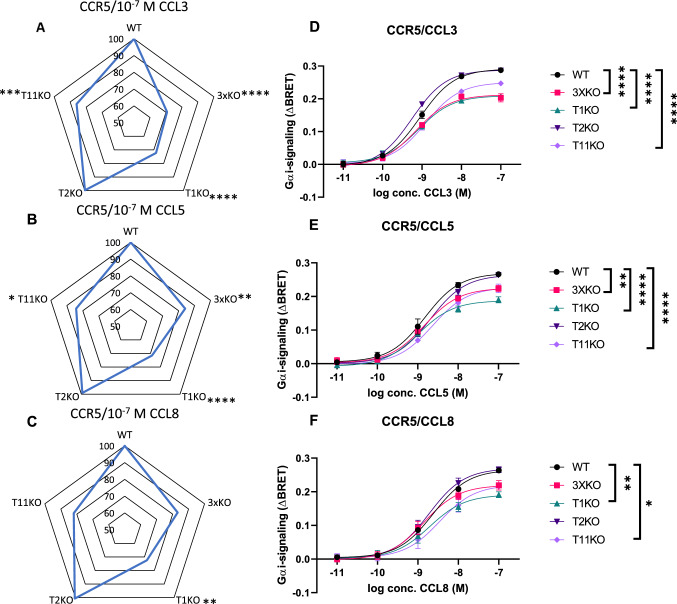
Analysis of CCR5-mediated signaling in Knock-out CHO cell lines affects cell signaling. Analysis of CHO cell lines transiently transfected with CCR5. Cells were stimulated with **A** CCL3, **B** CCL5 or **C** CCL8. The radar plots show the mean ΔBRET obtained in mutant CHO cell lines compared to mean obtained in the CHO WT cell line (normalized to 100%) in percentages of 3 independent experiments performed in duplicates. Statistically significant differences (*p* < 0.05) in comparison to WT for 10^–7^ M chemokine are indicated with an * for CCR5. **D**–**F** Cells were stimulated with **D** CCL3, **E** CCL5 or **F** CCL8. The results show the mean ΔBRET ± standard error of the mean (SEM) of 3 independent experiments performed in duplicate, 40 min after the addition of the ligand. Statistically significant differences by a two-way ANOVA test and a multiple comparison Tukey test between WT and another cell line are highlighted for the whole curve with stars: ****p-value < 0.0001, ***p-value ≤ 0.001, **p-value ≤ 0.01 and *p-value ≤ 0.05. Legend of symbols: Yellow square: GalNAc, yellow circle: galactose, purple triangle: sialic acid

The clear reduction of signaling in the 5XKO suggests that one or several of the GalNAc-Ts in this cell line can O-glycosylate CCR5. These 5 candidates are GalNAc-T1, -2, -7, -10 and -11. We, therefore, probed the signaling with a combination of GalNAc-T knock-out cell lines, to narrow down which enzymes are involved. We transfected CCR5 into the 3XKO (T1, 2 and 11 KO) and individual knock-outs for GalNAc-T1, 2 and 11. 3XKO CHO cells showed a clear decrease in signaling, suggesting that T1, T2 or/and T11 are important. Individual knock-out of T1 results in a clear decrease, whereas the T11KO cells show a more limited reduction and T2KO cells did not affect the signaling at all (Fig. 7, Fig. S8 and Tables S5, 6). Consequently, GalNAc-T1 is the most likely candidate for directly glycosylating CCR5 although T11 may also be involved.

### GalNAc-T1 and 11 can glycosylate an N-terminal CCR5 peptide in vitro

**Fig. 8:**
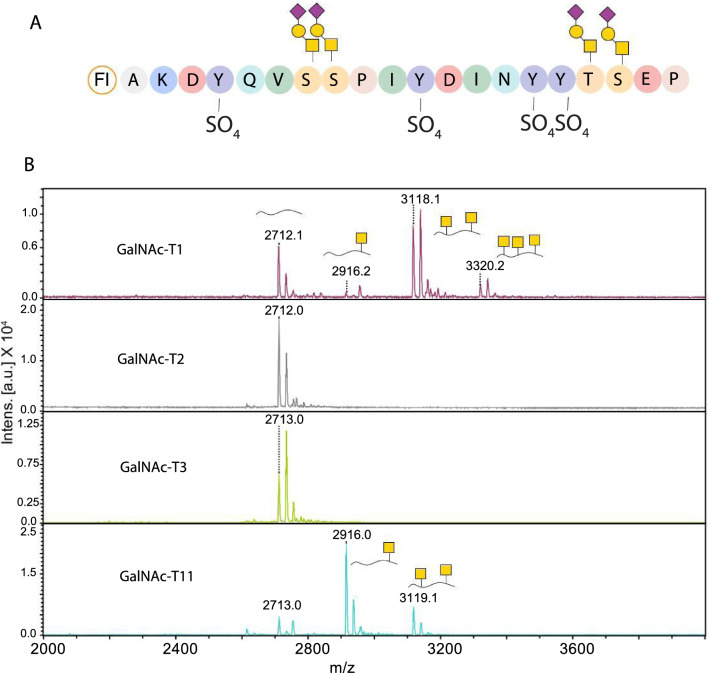
Analysis of GalNAc-T specificity towards the N-terminus of CCR5. **A** Sequence of the designed and unmodified peptide with amino acids shown with Rasmol colours based on their properties. Potential sites of O-glycosylation are shown with a core-1 structure and potential tyrosine sulfation is shown by a sulfate group. **B** A 20 mer peptide corresponding to the human CCR5 N-terminus was incubated with different recombinant GalNAc-Ts at 37 °C for overnight reaction. Samples were analyzed using MALDI-TOF and show that GalNAc-T1 was able to glycosylate the peptide at up to three different positions, whereas GalNAc-T11 could glycosylate only at two positions, while GalNAc-T2 and GalNAc-T3 failed to glycosylate the CCR5 peptide

The complete O-glycoproteome of a given cell is determined by its repertoire of *GALNT* genes coding for the different GalNAc-Ts. Some glycosylation sites are redundant, i.e., glycosylated by several enzymes, whereas other sites are controlled by a single GalNAc-T isoform. Some enzymes, as T1 and T2, have broader specificities and have major contributions to the total glycoproteome of a cell, whereas others such as T11 are much more restricted [8]. To test if some of our candidates GalNAc-Ts are able to directly glycosylate the N-terminus of CCR5, an in vitro glycosylation assay monitored by MALDI-TOF was performed. Briefly, custom synthesized CCR5 peptide corresponding to the CCR5 N-terminal region was incubated with recombinant human GalNAc-Ts and the addition of GalNAc was followed over time by MALDI-TOF analysis. As shown in Fig. 8 GalNAc-T1 and GalNAc-T11 were able to glycosylate the CCR5 peptide (*m*/*z* = 2712) at, respectively, three and two different sites, whereas GalNAc-T2 and GalNAc-T3 did not glycosylate the sequence. One GalNAc increases the *m*/*z* value by around 203. Indicating that the extra peaks found after incubation by GalNAc-T1 and GalNAc-T11 correspond to multiple glycosylated sites. A MUC1-derived peptide was used as a positive control for the activity of the enzymes due to its many glycosylation sites and as such being a substrate for almost all GalNAc-Ts (Fig. S5) [44]. These results further underline the involvement of GalNAc-T1 and GalNAc-T11 and show that the functional implications that we observe in the signaling are likely a result of glycosylation in the CCR5 N-terminus.

### Negative charges added through glycosylation and sulfation may cooperate in fine-tuning chemokine binding and downstream signaling

**Fig. 9:**
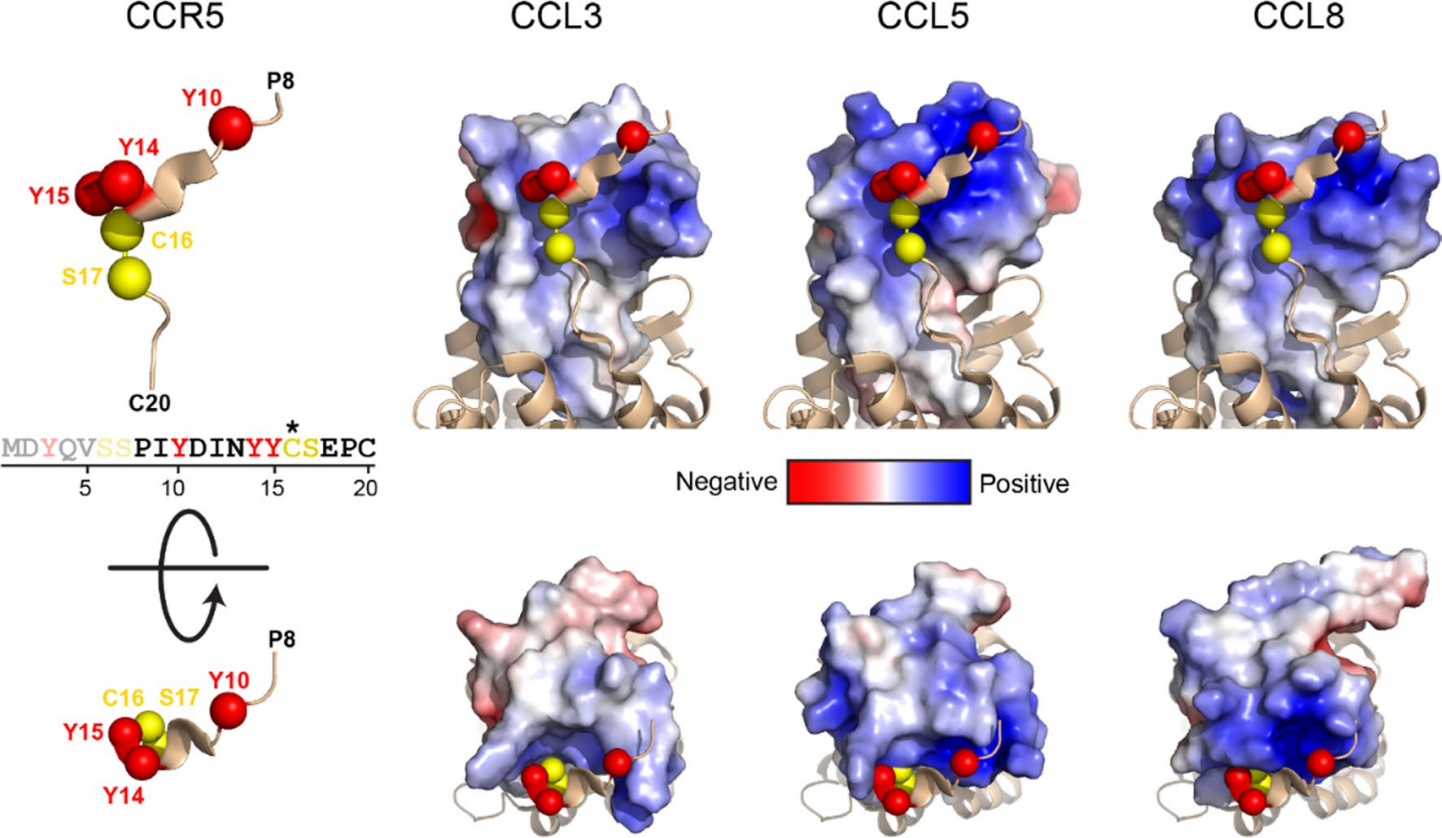
Possible glycosylation sites and residues susceptible to tyrosine sulfation on crystal and NMR structures. Location of glycosylated and sulfated residues in CCR5:chemokine complexes. Potentially sulfated and glycosylated residues are highlighted with red and yellow spheres, respectively. CCL3 (PDB ID: 5COR), CCL5 (5COY) and CCL8 (1ESR) are colored by electrostatic potential and aligned with CCL3 in the CCR5:CCL3 complex (7F1T). Top and bottom panels show side and top views, respectively. The Cys residue at position 16 is highlighted as a glycosylation site since the native sequence has a Thr residue in this position. The first seven residues of CCR5 are not shown due to a lack of electron density from the X-ray data

The negative charge of the chemokine receptor N-termini is well known to be an important determinant of chemokine binding and discrimination. Similarly, the role of glycosylation and sulfation in this N-terminal region has been described for a number of chemokine receptors [4], but it is still not understood how these affect each other. To visualize the importance of the glycosylation and sulfation for chemokine binding, available crystal and NMR structures of chemokines bound to CCR5 were used (Fig. 9). Glycan structures can vary substantially in size and branching and/or (poly)sialylation may have a huge impact on the spatial impact and effect on the binding on other PTMs. Without addressing this complexity this analysis illustrates well how sulfation and glycosylation are in close proximity to the positively charged regions of the chemokines. CCL5 and CCL8 contain more positive charges compared to CCL3, which may explain the different effects observed during signaling assays. Removal of the negatively charged terminal sialic acid(s) may affect CCL5 and CCL8 more drastically due to this change in charge, making them potentially less able to recognize/bind to CCR5. CCL3, which is less charged may then have less problems in recognition and/or binding. This suggests the importance of O-glycosylation and tyrosine sulfation in the fine-tuning of chemokine ligand and receptor binding.

## Discussion

In silico analyses suggests that CC chemokine receptors have general patterns of O-glycosylation and tyrosine sulfation in their N-terminal region. A growing amount of evidence proposes that both PTMs are important for chemokine binding and signaling, but also that the effect is complex and potentially differs between receptor-ligand pairs. We have only just begun to scratch the surface regarding the combinatorial effect of modifications and the effect of specific acceptor sites and glycan compositions. Here, we have used engineered cell lines, inhibitors and a mutational approach to probe the effect on chemokine signaling through CCR1 and CCR5 when changing O-glycosylation and tyrosine sulfation. Our findings point to the importance of the negative charges provided by these PTMs for the binding of CCL5 and CCL8 and to a minor extend CCL3. Additionally, we find that (1) changing tyrosine sulfation alters the impact of O-glycosylation considerably; (2) the presence of O-glycans on the chemokine receptor affects the potency and efficacy of CCR5-mediated Gα_i_-signaling and (3) removal of the terminal sialic acid is sufficient to affect the signaling. Sialic acids are negatively charged and are likely important in the recognition of CCL5 and CCL8 by interacting with their positive residues and a similar effect has been reported previously [14]. Expression of PolySia does not by itself affect the CCR5 signaling but only in the context of an abolished tyrosine sulfation status. Tyrosine sulfation was removed by either NaClO_3_ or by mutating the acceptor sites. Both approaches almost completely abolished Gα_i_-signaling. However, a notable difference was that mutation of the four tyrosine residues to phenylalanine also led to the loss of O-glycosylation, whereas this was not the case for cells treated with NaClO_3_. This could be because altering the primary sequence of CCR5 disrupts the recognition of responsible GalNAc-Ts.

The reduction in the CCR5-mediated Gα_i_-signaling was very similar with either method to remove tyrosine sulfation, and expression of PolySia was also able to partially rescue the signaling in both cases. This suggests that tyrosine sulfation is essential for general signaling whereas O-glycosylation is more important as a fine-tuner. Furthermore, the effect of PolySia is presumably not through CCR5 O-glycosylation, as we still observed the effect in the mutated sequence which had lost the O-glycosylation and the mobility of CCR5 in western blot analysis was also not affected in the PolySia expressing cells. How PolySia exerts its compensatory effect is not clear, but it may be through specific surface molecules carrying this modification. The best-described carrier of polysialylation is the neural cell adhesion molecule (NCAM) which is important for neural development and plasticity [45]. In addition, polysialylation of the O-glycan on Neuropilin-2 (NRP2), a co-receptor for vascular endothelial growth factors, mediates CCL21-driven chemotaxis of dendritic cells (DC). The N- and O-glycans of chemokine receptor CCR7 can also carry polysialic acids and this affects the recognition of the chemokine CCL21 and subsequent dendritic cell migration [10]. A similar mechanism involving a polysialylated co-receptor could be involved in the case of CCL5 and CCL8. However, it is also possible that the effect is not driven by a specific carrier. It can be caused by the increased general negative charge of the cell surface and more comparable to the effect of glycosaminoglycans which are important for forming chemokine gradients and oligomerization [46, 47].

ST8SIA4 is a polysialyltransferase which is responsible for the linkage of α2-8-glycosidically linked homopolymers of sialic acids attached to N- and/or O-glycans [10, 45]. It has been identified to be associated with systemic lupus erythematosus (SLE) in a genome-wide association study [48]. *T*-cells are central in SLE pathogenesis and expression of CCR1 and CCR5 has been linked to disease progression although with contradictory findings between the protective versus damaging effect of both CCR5 [49–51] and the CCR5-delta32 variant with a truncated N-terminus [50]. These reports could suggest that the PTMs of CCR5 (and the immune cells expressing it) may also need to be considered to understand the association and progression of SLE in this context.

Up to twenty different GalNAc-Ts can initiate O-glycosylation and the substrate specificities of the various GalNAc-Ts are still not completely understood. However, certain isoforms, such as GalNAc-T1, T2 and T3 are generally known to have many substrates and are widely expressed [52]. Other sites are more specifically controlled, such as the GalNAc-T11 specific sites in the low-density lipoprotein receptor (LDLR) family [53, 54]. Understanding, the specificity of individual sites is not trivial and may depend on several cell-specific determinants including the GalNAc-T repertoire. Using recombinantly expressed enzymes for in vitro glycosylation may provide clues to the specificities but does not completely reflect the in vivo specificities [55] and enzyme levels also affect the glycosylation status of individual sites [56]. The O-glycosylation of CCR5 may, therefore, vary significantly between different tissues and cell types or even the maturation status of individual immune cell subtypes. Taken together it is a difficult task to identify responsible GalNAc-Ts for CCR5 O-glycosylation in vivo*.* However, with the available methods, we identified likely candidates, and several CHO GalNAc-T knock-outs transfected with CCR5 were tested and a significant negative effect on signaling was found when cells were lacking either T1 or T11. We also performed in vitro glycosylation of the N-terminal sequence and found T1 and T11 to be able to glycosylate the sequence, whereas GalNAc-T2 and GalNAc-T3 could not. T1 is widely expressed including in the immune system, and a previous study found that GALNT1 KO mouse have impaired leukocyte recruitment, which may be due to the lack of PSGL-1 glycosylation [57]. The chemokine signaling was not investigated in this study, and together with our observations, this points to GalNAc-T1 as being the most interesting isoform for future follow-up studies aiming at our understanding of the regulation and initiation of CCR5 O-glycosylation.

The “glyco-sulfo barcodes” will inevitably vary in different cell types and tissues. Glycosyltransferase repertoires differ in recognition of both glycan sites and structures. The combinatorial space is huge when multiple sites of both PTM types in a single receptor N-terminus are considered, which for CCR5 are 4 potential sulfosites and 4 potential O-glycosylation sites. It remains to be discovered how much of these details matter for basic biology and how we can potentially utilize this in future drug design. There may be redundancy between the many possible patterns and some sites could serve more critical or specific functions. As was recently described, a single O-glycosylation site in the N-terminus of CCR7 seems to be required for boosting the effect of CCR7 functioning by C-terminal peptide fragments of CCL21 [16]. We need more information on the biological importance of specific sites and structures and a continuous improvement of current methods is key.

Glycoengineering of cell lines has provided new possibilities for probing glycosylation differences. One can argue that this dissection needs to be performed in relevant cells expressing the chemokine receptor endogenously together with relevant enzymes and co-receptors. However, even if this was currently feasible, we still cannot rule out the indirect effects of removing one enzyme since many glycosyltransferases and the two TPSTs also carry O-glycosylation [58]. Moreover, tyrosine sulfation can differ between cell lines or even on the same cell [23]. A combination of approaches including genetic engineering and acceptor site mutation are important complementary methods to pinpoint the important determinants.

One important perspective is the possibility of utilizing glyco-sulfo patterns for novel drug design. PSGL-1 glycosulfo peptide analogue GSnP-6 was previously demonstrated to display nanomolar affinity and promising potential for blocking PSGL-1/P-selectin interaction [59]. Sulfated mCCR2 peptides can decrease retinal degradation in mice by competing for available chemokines and thereby potentially reduce immune cell recruitment [60] and the use of the chemokine binding tick-derived evasins has been suggested as an untapped resource for novel targeting of the immune system [61]. Moreover, different antibodies have shown distinct affinities for individual sulfo-forms of CCR5 [23]. Consequently, a more comprehensive understanding of the glycosylation and sulfation patterns of CCRs may allow for new precise targeting of the immune system either through using modifications as “anchors” thereby targeting subsets of receptors or by directly mimicking the code by peptides all small molecules.

We have only started to learn how O-glycosylation and tyrosine sulfation regulate the chemokine system. Further research will be needed to obtain a precise understanding of the glyco-sulfo barcodes on the chemokine receptors and other GPCRs and thereby the role of these PTMs in for instance the complex interaction and promiscuity of the chemokine system. As of now, CCR1 and CCR5 are shown to be affected by O-glycosylation and tyrosine sulfation in the fine-tuning and recognition of chemokines and the subsequent effect of receptor signaling and functioning.

## Supplementary Information

Below is the link to the electronic supplementary material.

Supplementary file1 (PPTX 130 KB)

Supplementary file2 (PPTX 168 KB)

Supplementary file3 (PPTX 4954 KB)

Supplementary file4 (PPTX 602 KB)

Supplementary file5 (PPTX 130 KB)

Supplementary file6 (DOCX 805 KB)

## Data Availability

This study includes no data deposited in external repositories.
