# Supplementary material for: “Glyco-sulfo barcodes” regulate chemokine receptor function
Source: Cell Mol Life Sci. 2023 Feb 2;80(2):55. doi: 10.1007/s00018-023-04697-9 (PMC9894980; doi:10.1007/s00018-023-04697-9)
Supplement: Supplementary file 1 — Supplementary file1 (PPTX 130 KB) [file 18_2023_4697_MOESM1_ESM.pptx]

## Slide 1
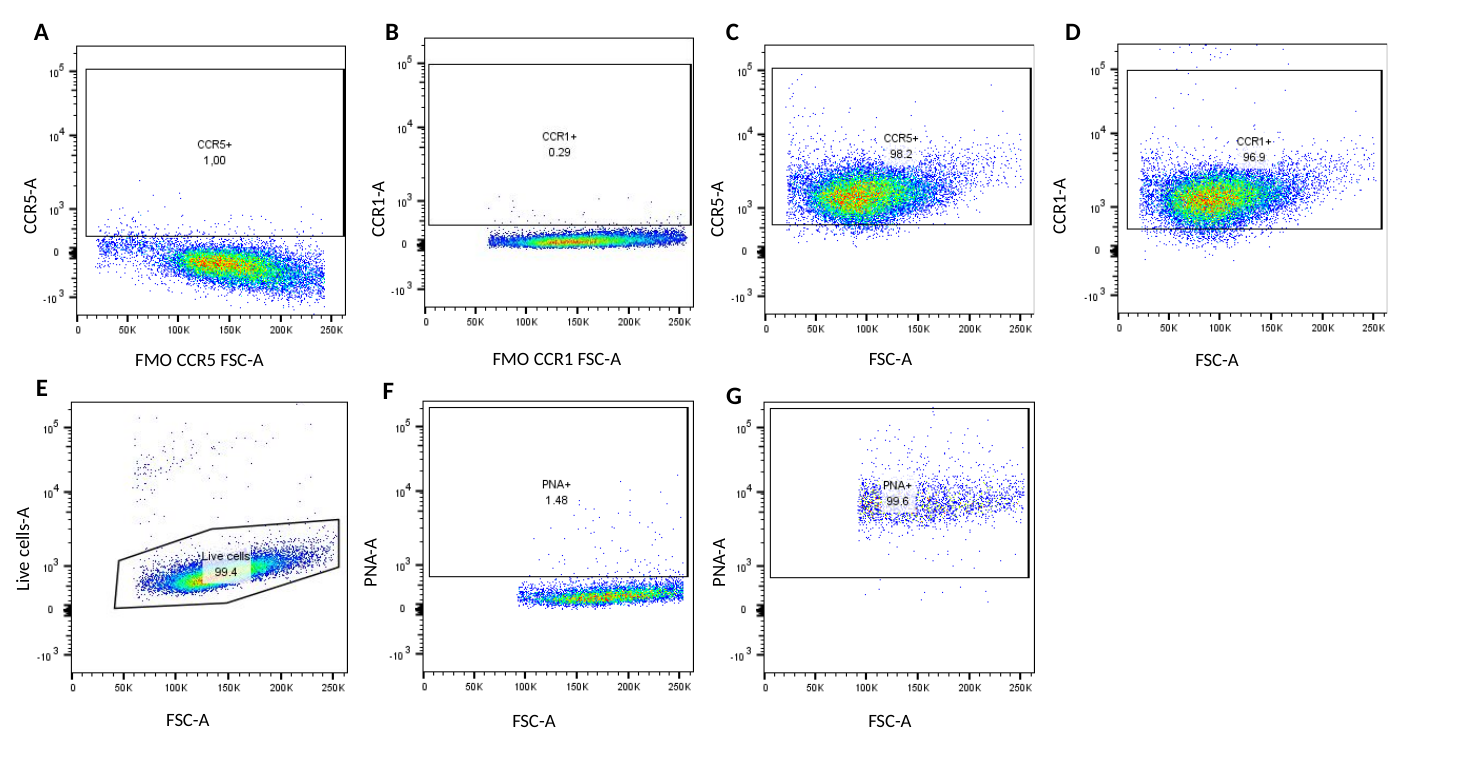

D
C
A
B
CCR1-A
CCR1-A
CCR5-A
CCR5-A
FMO CCR1 FSC-A
FSC-A
FMO CCR5 FSC-A
FSC-A
E
F
G
PNA-A
PNA-A
 Live cells-A
FSC-A
FSC-A
FSC-A
