# Supplementary figures and images for: “Glyco-sulfo barcodes” regulate chemokine receptor function
Source: Cell Mol Life Sci. 2023 Feb 2;80(2):55. doi: 10.1007/s00018-023-04697-9 (PMC9894980; doi:10.1007/s00018-023-04697-9)

## Slide 1
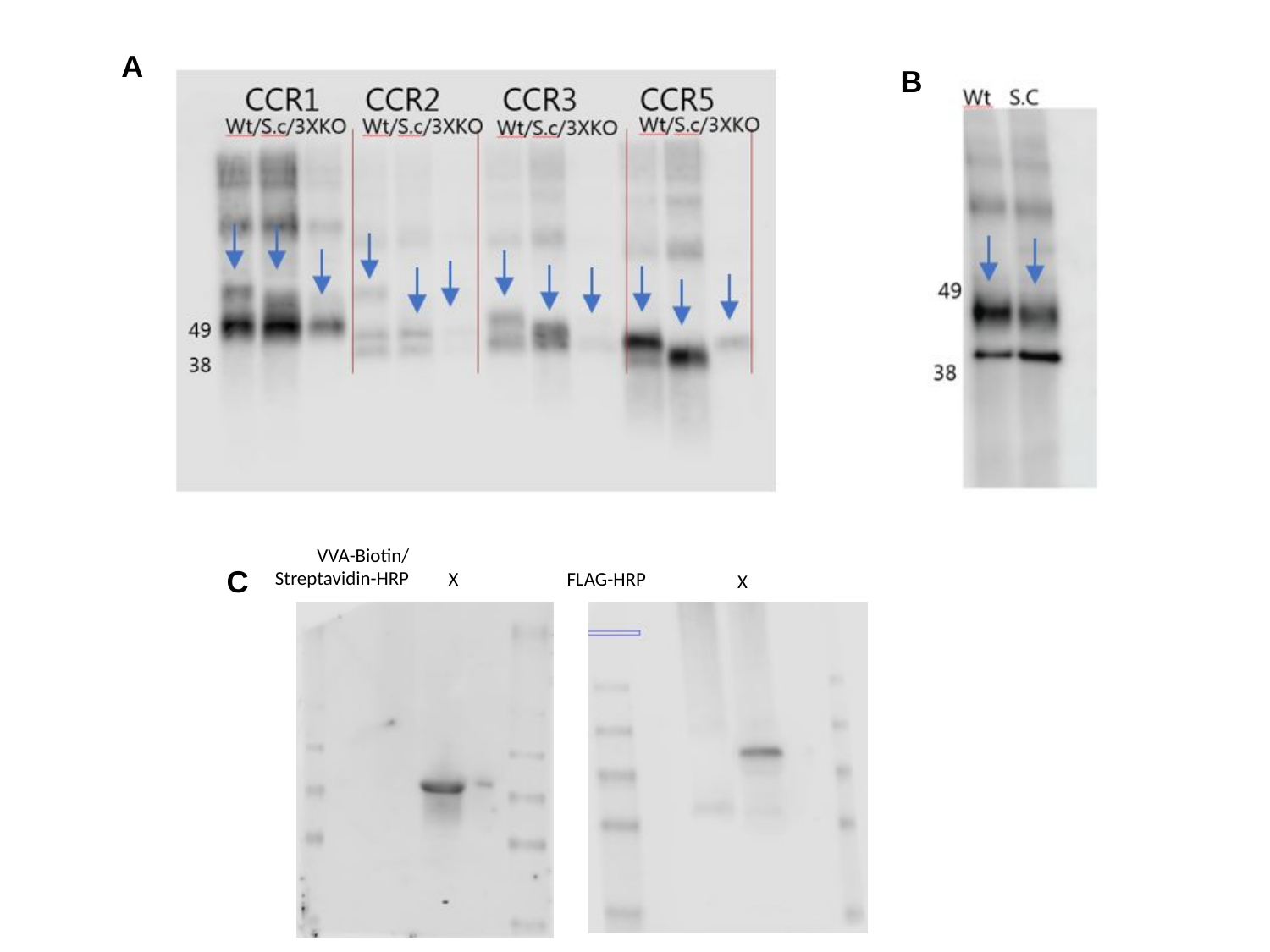

A
B
VVA-Biotin/
Streptavidin-HRP
C
X
FLAG-HRP
X

Supplement: Supplementary file 2 — Supplementary file2 (PPTX 168 KB) [file 18_2023_4697_MOESM2_ESM.pptx]

## Slide 1
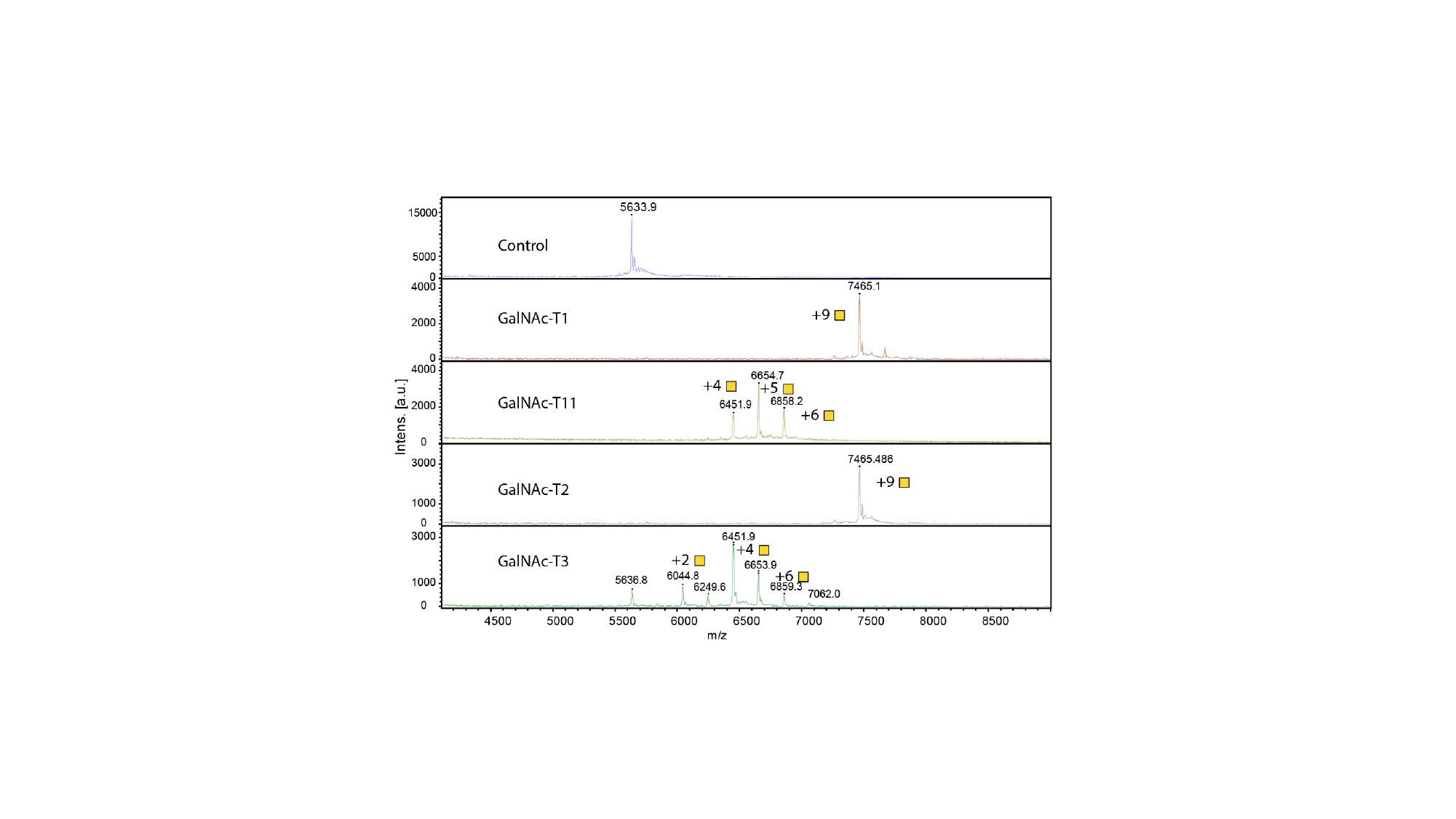

Supplement: Supplementary file 5 — Supplementary file5 (PPTX 130 KB) [file 18_2023_4697_MOESM5_ESM.pptx]
