# Supplementary material for: “Glyco-sulfo barcodes” regulate chemokine receptor function
Source: Cell Mol Life Sci. 2023 Feb 2;80(2):55. doi: 10.1007/s00018-023-04697-9 (PMC9894980; doi:10.1007/s00018-023-04697-9)
Supplement: Supplementary file 3 — Supplementary file3 (PPTX 4954 KB) [file 18_2023_4697_MOESM3_ESM.pptx]

## Slide 1
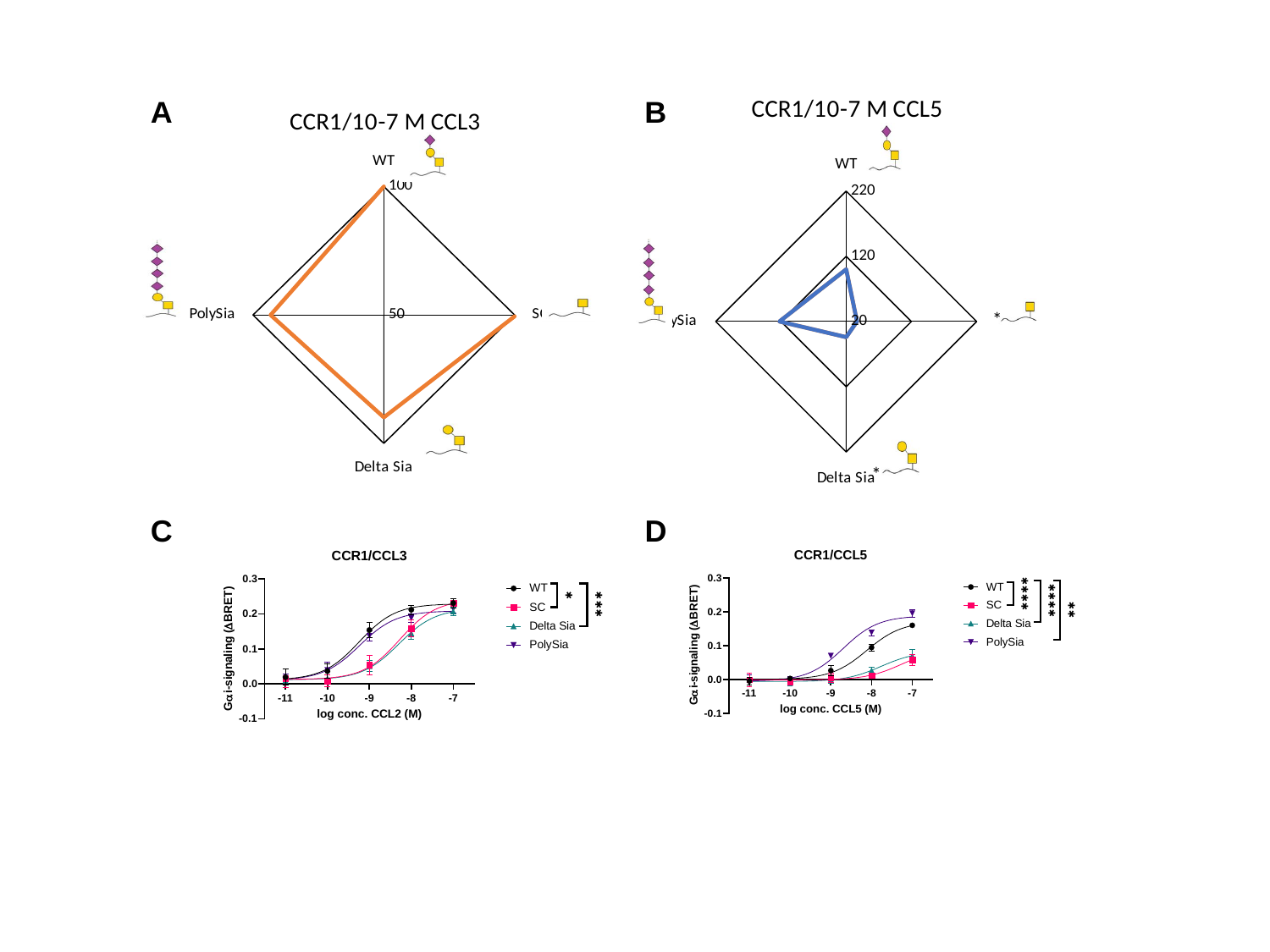

### Chart: CCR1/10-7 M CCL5
| Category | CCR1 | CCR1 |
|---|---|---|
| WT | 100.0 | 100.0 |
| SC | 36.67465644806563 | 36.67465644806563 |
| Delta Sia | 44.052327002168305 | 44.052327002168305 |
| PolySia | 122.22764117735805 | 122.22764117735805 |
### Chart: CCR1/10-7 M CCL3
| Category | CCR1 |
|---|---|
| WT | 100.0 |
| SC | 100.06976051295229 |
| Delta Sia | 89.88220068086808 |
| PolySia | 93.25588257148635 |C
D
