# Supplementary material for: “Glyco-sulfo barcodes” regulate chemokine receptor function
Source: Cell Mol Life Sci. 2023 Feb 2;80(2):55. doi: 10.1007/s00018-023-04697-9 (PMC9894980; doi:10.1007/s00018-023-04697-9)
Supplement: Supplementary file 4 — Supplementary file4 (PPTX 602 KB) [file 18_2023_4697_MOESM4_ESM.pptx]

## Slide 1
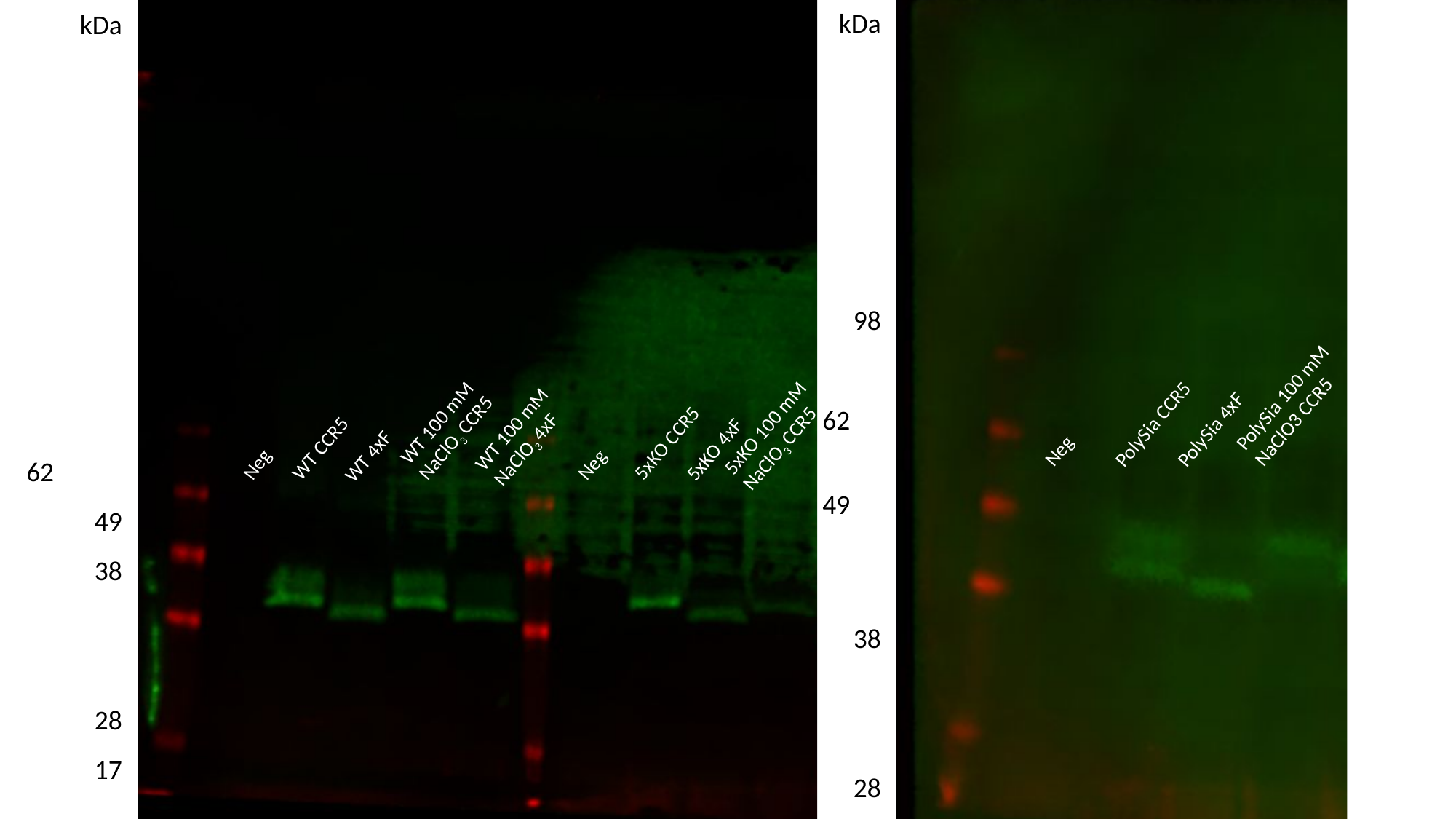

kDa
62
 49
 38
28
17
kDa
98
62
49
38
28
17
Neg
PolySia 100 mM NaClO3 CCR5
WT 100 mM NaClO3 CCR5
WT 100 mM NaClO3 4xF
Neg
PolySia 4xF
PolySia CCR5
5xKO 100 mM NaClO3 CCR5
Neg
WT CCR5
Neg
5xKO CCR5
5xKO 4xF
WT 4xF
