# Supplementary material for: “Glyco-sulfo barcodes” regulate chemokine receptor function
Source: Cell Mol Life Sci. 2023 Feb 2;80(2):55. doi: 10.1007/s00018-023-04697-9 (PMC9894980; doi:10.1007/s00018-023-04697-9)
Supplement: Supplementary file 6 — Supplementary file6 (DOCX 805 KB) [file 18_2023_4697_MOESM6_ESM.docx]

**Supplementary Information**

**“Glyco-Sulfo Barcodes” Regulate Chemokine Receptor Function**

Cellular and Molecular Life Sciences,

Lisa Verhallen^1,2^, Jarkko J. Lackman^1^, Rikke Wendt^1^_,_ Martin Gustavsson^1^, Zhang Yang^3^, Yoshiki Narimatsu^3^, Daniel M. Sørensen^3^, Kato Mac Lafferty^2^, Mieke Gouwy^2^, Pedro E. Marques^2^ Gertrud M. Hjortø^1^, Mette M. Rosenkilde^1^, Paul Proost^2^ and Christoffer K. Goth^1*^

Corresponding author: Christoffer K. Goth

University of Copenhagen

Department of Biomedical Sciences

Lab for Molecular Pharmacology

Panum building 18,5. Blegdamsvej 3B, Copenhagen N, DK-2200

Tel: +4561745850

Email:  [goth@sund.ku.dk](mailto:goth@sund.ku.dk)

**Fig. S1.**

**
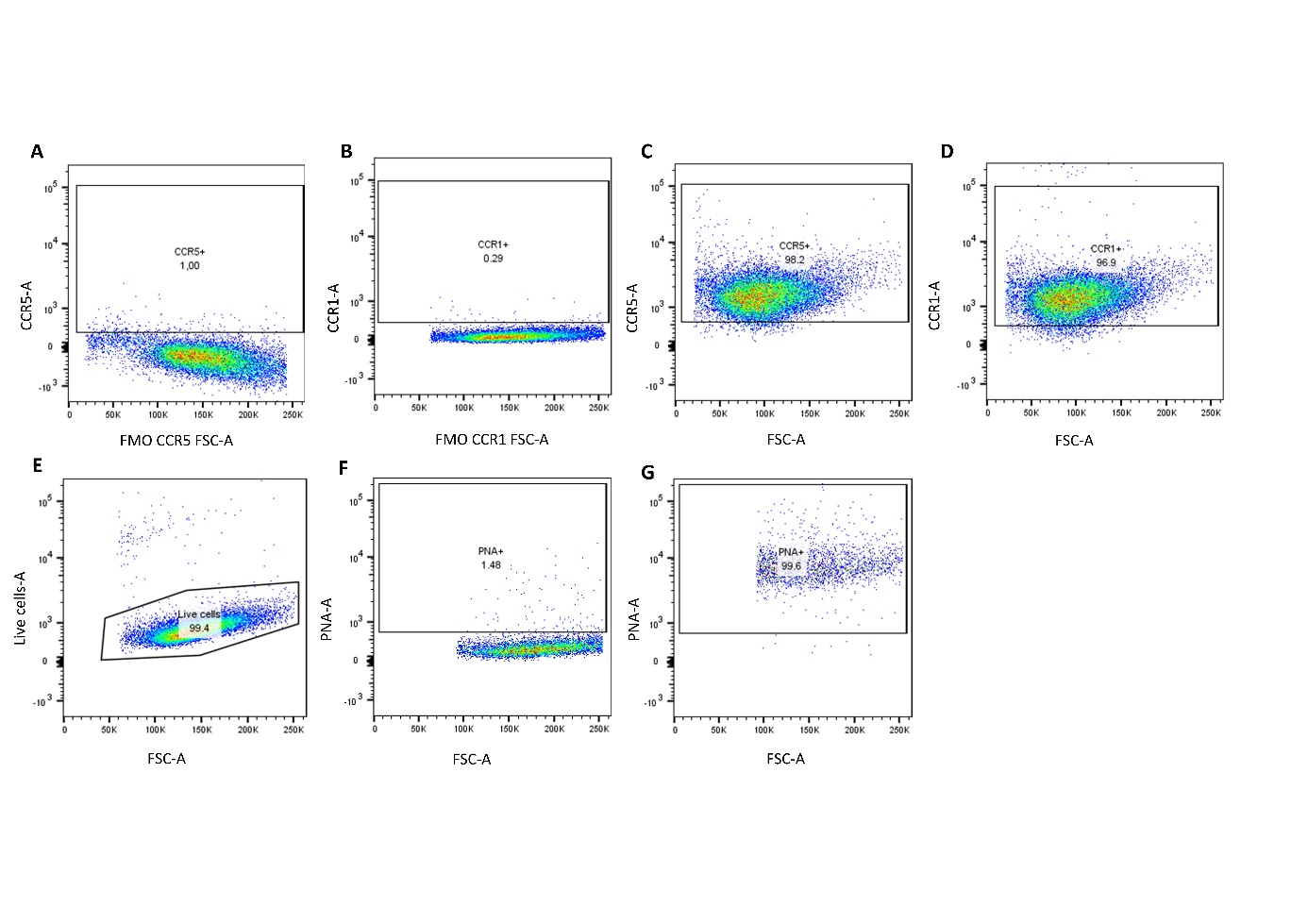
**

**Example of FMO and flow cytometry plots**

**A-B** Example of FMO controls used to determine **A** CCR5 and **B** CCR1 expression. **C-E** Examples of flow cytometry plots showing **C** CCR5, **D** CCR1 expression and **E** the amount of live cells of 1 experiment. **F-G** Flow cytometry graphs showing the lectin PNA expression with recognizes Core 1 O-glycans without sialic acids of **F** THP-1 cell not treated with neuraminidase and **G** THP-1 cells treated with neuraminidase.

**Fig. S2**

**
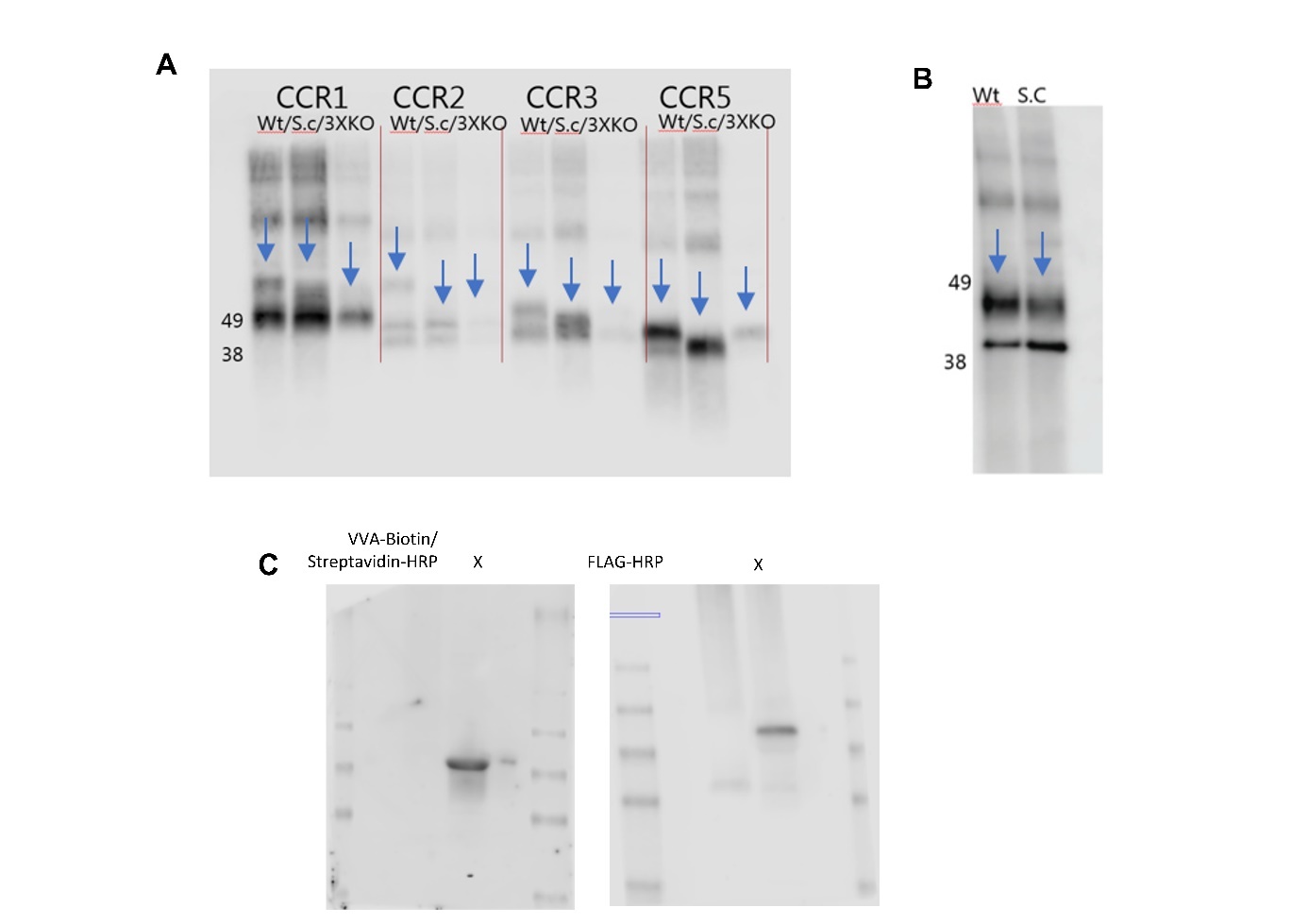
**

**Western blot of immunoprecipitated samples from transfected HEK cells.**

**A** Transiently transfected WT, SC and 3XKO HEK cells were lysed, immunoprecipitated and analyzed by western blot. All receptors CCR1, CCR3 and CCR5 showed a decrease in molecular mass when expressed in SC. Whereas CCR1, CCR2 and CCR3 also showed a decreased molecular mass in 3XKO (simultaneous T1, T2 and T3 KO), CCR5 did not, suggesting that remaining GalNAc-T(s) are active in this cell line. **B** CHO cells were transiently transfected with CCR2 and analyzed by immunoprecipitation and western blot. No shift was observed between the WT and “simple cells (SC), suggesting that CCR2 is not O-glycosylated in CHO cells. **C** Full western blots of fig 3B.

**Fig. S3**

**
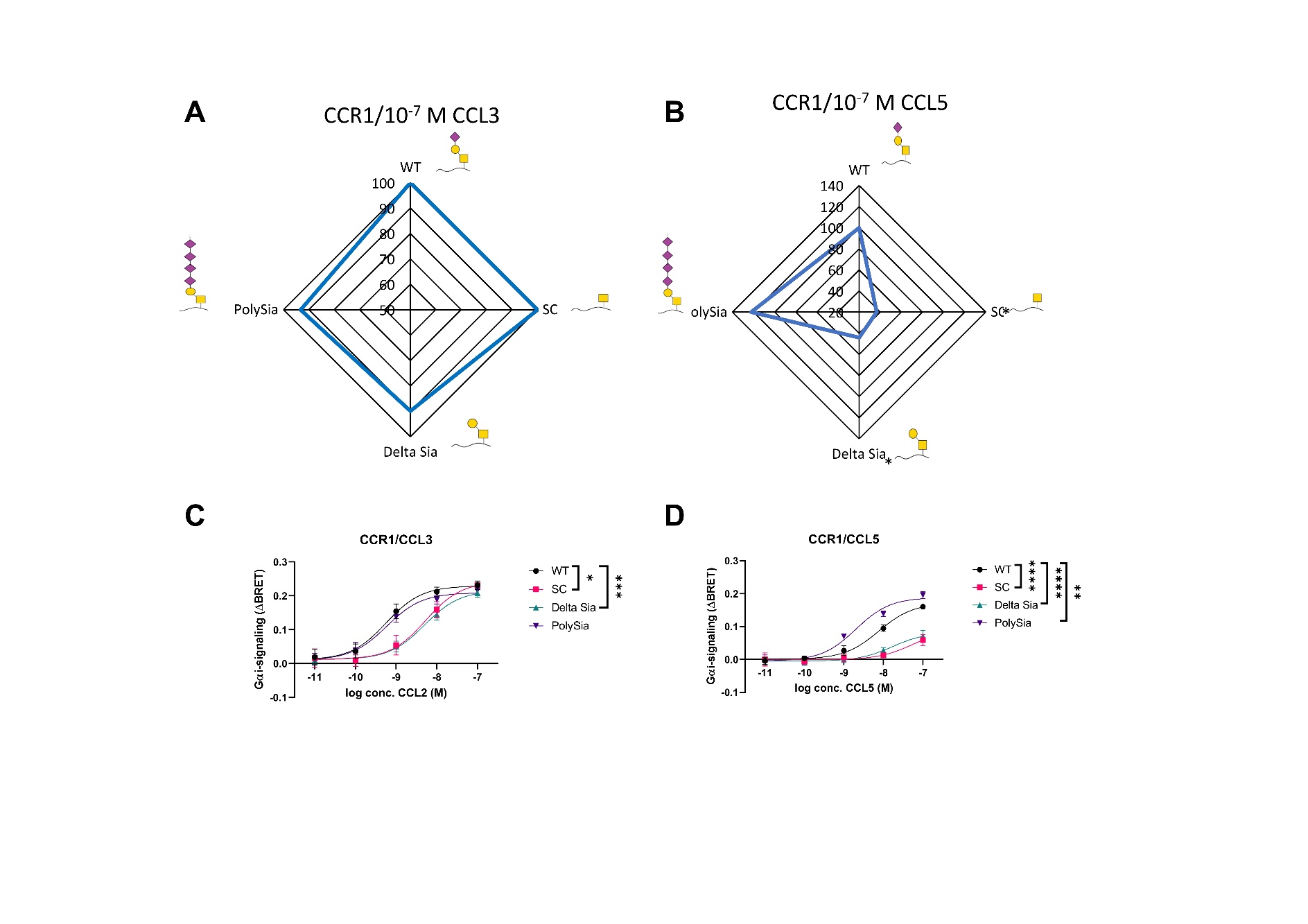
**

**Analysis of CHO cell lines transiently transfected with CCR1**

**A-B** Cells were stimulated with 100 nM **A** CCL3 or **B** CCL5. The radar plots show the mean ΔBRET compared to WT (normalized to 100 percent) in percentages of 3 independent experiments, performed in duplicate, 40 minutes after addition of the ligand. Statistical analysis of differences in signaling and complete dose-response curves can be found in Appendix table S1 and S2 and Appendix figure S5. Statistically significant differences (p<0.05) in comparison to WT for 10^-7^ M chemokine are indicated with an *. **C-D** Cells were stimulated with **C** CCL3 or **D** CCL5. The results show the mean ΔBRET + standard error of the mean (SEM) of three independent experiments performed in duplicate 40 minutes after addition of the ligand. Statistically significant differences by a two-way ANOVA test and a multiple comparison Tukey test between WT and another cell line are highlighted for the whole curve with stars: ****p-value<0.0001, ***p-value≤0.001 and *p-value ≤0.05

**Table S1**

| **Chemokine receptor** | **Chemokine** | **Cell line** | **EC50 (nM)** | **95% confidence interval of the EC50 (nM)** |
| --- | --- | --- | --- | --- |
| CCR1 | CCL3 | WT | 0.57 | [0.22, 1.40] |
|  |  | SC | 5.34 | [1.57, 16.26] |
|  |  | Delta Sia | 5.02 | [2.19, 10.70] |
|  |  | PolySia | 0.58 | [0.22, 1.45] |
|  | CCL5 | WT | 7.64 | [3.44, 15.70] |
|  |  | SC | 53.21 | [1.64, -] |
|  |  | Delta Sia | 17.09 | [3.21, -] |
|  |  | PolySia | 2.01 | [0.95, 4.98] |
| CCR5 | CCL3 | WT | 1.01 | [0.36, 2.89] |
|  |  | SC | 2.05 | [0.70, 6.89] |
|  |  | 5xKO | 1.43 | [0.62, 3.50] |
|  |  | T3KI | 0.89 | [0.42, 1.85] |
|  |  | Delta Sia | 2.00 | [0.57, 3.45] |
|  |  | PolySia | 0.57 | [0.19, 1.15] |
|  | CCL5 | WT | 0.58 | [0.28, 1.10] |
|  |  | SC | 1.71 | [0.30, 12.28] |
|  |  | 5xKO | 1.08 | [0.63, 1.87] |
|  |  | T3KI | 0.61 | [0.29, 1.20] |
|  |  | Delta Sia | 1.34 | [0.57, 3.45] |
|  |  | PolySia | 0.49 | [0.19, 1.15] |
|  | CCL8 | WT | 2.36 | [1.64, 3.45] |
|  |  | SC | 10.40 | [7.98, 13.54] |
|  |  | 5xKO | 4.23 | [2.58, 6.78] |
|  |  | T3KI | 3.72 | [2.72, 6.78] |
|  |  | Delta Sia | 6.19 | [4.23, 8.87] |
|  |  | PolySia | 1.12 | [0.58, 2.22] |

**EC50 value for experiments evaluating O-glycosylation**

The 95% confidence interval of the EC50 values based on the dose-response curves of the CCR1/CCR5-mediated Gα_i_-signaling induced by CCL3, CCL5 or CCL8 in the different CHO cell lines.

**Table S2**

| **Receptor** | **Chemokine** | **Cell lines compared** | | **P-value** | |
| --- | --- | --- | --- | --- | --- |
| CCR1 | CCL5 | WT | SC | <0.0001 | **** |
|  |  |  | Delta Sia | <0.0001 | **** |
|  |  | SC | PolySia | <0.0001 | **** |
|  |  | Delta Sia | PolySia | <0.0001 | **** |
| CCR5 | CCL5 | WT | SC | 0.0026 | ** |
|  |  |  | 5XKO | 0.0250 | * |
|  |  |  | Delta Sia | <0.0001 | **** |
|  |  | SC | PolySia | 0.0373 | * |
|  |  | T3KI | Delta Sia | 0.0370 | * |
|  |  | Delta Sia | PolySia | 0.0012 | ** |
|  | CCL8 | WT | SC | 0.0010 | *** |
|  |  |  | 5XKO | <0.0001 | **** |
|  |  |  | Delta Sia | <0.0001 | **** |
|  |  | SC | Delta Sia | 0.0140 | * |
|  |  |  | PolySia | 0.0276 | * |
|  |  | 5XKO | T3KI | 0.0162 | * |
|  |  |  | PolySia | <0.0001 | **** |
|  |  | T3KI | Delta Sia | <0.0001 | **** |
|  |  | Delta Sia | PolySia | <0.0001 | **** |

**Statistical analysis for Gα_i_-signaling measured by BRET**

Statistical analysis was performed to determine significant differences in CCR1/CCR5-induced Gα_i_-signaling after activation by 100 nM CCL3, CCL5 or CCL8 between the cell lines. A two-way ANOVA test was performed, followed by a multiple-comparison Tukey test for the main column effects by computing the means of each data set column. P-values are only listed for the cell lines that showed statistically significant differences. The significance level is indicated by stars next to the p-value; ****p-value<0.0001, ***p-value<0.001, **p-value<0.01 and *p-value≤0.05.

**Table S3**

| **Chemokine receptor** | **Chemokine** | **Cell line** | **95% confidence interval of the EC50 (nM)** |
| --- | --- | --- | --- |
| CCR5 4xF | CCL3 | WT | [14.86, 104.90] |
|  |  | SC | [7.92, 183.80] |
|  |  | 5xKO | [48.21, 213.20] |
|  |  | T3KI | [12.30, 43.48] |
|  |  | Delta Sia | [18.39, 93.92] |
|  |  | PolySia | [6.29, 35.58] |
|  | CCL5 | PolySia | [5.27, 23.16] |
| CCR5 + 100 mM NaClO_3_ | CCL3 | WT | [4.37, 167.70] |
|  |  | 5xKO | [1.36, 362.40] |
|  |  | T3KI | [2.77, 20.47] |
|  |  | PolySia | [1.72, 21.15] |
|  | CCL5 | PolySia | [0.30, 19.13] |

**95% confidence intervals of the EC50 after removal of tyrosine sulfation**

The 95% confidence interval of the EC50 values based on the dose-response curves of the CCR5 Gα_i_-signaling induced by CCL3, CCL5 or CCL8 in the different CHO cell lines after removal of tyrosine sulfation. EC50 values are only given when it was possible to determine a 95% confidence interval.

**Table S4**

| **Receptor** | **Chemokine** | **Cell lines compared** | | **P-value** | |
| --- | --- | --- | --- | --- | --- |
| CCR5 4xF | CCL3 | 5XKO | WT | 0.0050 | ** |
|  |  |  | T3KI | 0.0091 | ** |
|  |  | PolySia | SC | <0.0001 | **** |
|  |  |  | 5XKO | <0.0001 | *** |
|  |  |  | Delta Sia | 0.0003 | *** |
|  | CCL5 | PolySia | WT | <0.0001 | **** |
|  |  |  | SC | <0.0001 | **** |
|  |  |  | 5XKO | <0.0001 | **** |
|  |  |  | T3KI | <0.0001 | **** |
|  |  |  | Delta Sia | <0.0001 | **** |
|  | CCL8 | PolySia | WT | <0.0001 | **** |
|  |  |  | SC | <0.0001 | **** |
|  |  |  | 5XKO | <0.0001 | **** |
|  |  |  | T3KI | <0.0001 | **** |
|  |  |  | Delta Sia | <0.0001 | **** |
| CCR5 + 100 mM NaClO_3_ | CCL3 | SC | T3KI | 0.0001 | *** |
|  |  |  | PolySia | 0.0054 | ** |
|  |  | 5XKO | WT | 0.0482 | * |
|  |  |  | T3KI | <0.0001 | **** |
|  |  |  | PolySia | 0.0003 | *** |
|  |  | T3KI | Delta Sia | 0.0047 | ** |
|  | CCL5 | SC | T3KI | 0.0296 | * |
|  |  | PolySia | WT | 0.0003 | *** |
|  |  |  | SC | <0.0001 | **** |
|  |  |  | 5XKO | <0.0001 | **** |
|  |  |  | T3KI | 0.0336 | * |
|  |  |  | Delta Sia | <0.0001 | **** |
|  | CCL8 | SC | WT | 0.0021 | ** |
|  |  |  | 5XKO | 0.0354 | * |
|  |  |  | Delta Sia | 0.0373 | * |
|  |  | T3KI | WT | <0.0001 | **** |
|  |  |  | SC | <0.0001 | **** |
|  |  |  | 5XKO | <0.0001 | **** |
|  |  |  | Delta Sia | <0.0001 | **** |
|  |  |  | PolySia | <0.0001 | **** |
|  |  | PolySia | WT | <0.0001 | **** |
|  |  |  | SC | <0.0001 | **** |
|  |  |  | 5XKO | <0.0001 | **** |
|  |  |  | Delta Sia | <0.0001 | **** |

**Statistical analysis for Gαi-signaling measured by BRET after removal of tyrosine sulfation**

Statistical analysis performed to determine significant differences in CCR5-induced Gα_i_-signaling between the cell lines after activation with 100 nM CCL3, CCL5 or CCL8. A two-way ANOVA test was performed, followed by a multiple-comparison Tukey test for the main column effects by computing the means of each data set column. P-values are only listed for the cell lines that showed statistically significant differences. The significance level is indicated by stars next to the p-value; ****p-value<0.0001, ***p-value<0.001, **p-value<0.01 and *p-value ≤0.05.

**Fig. S4**


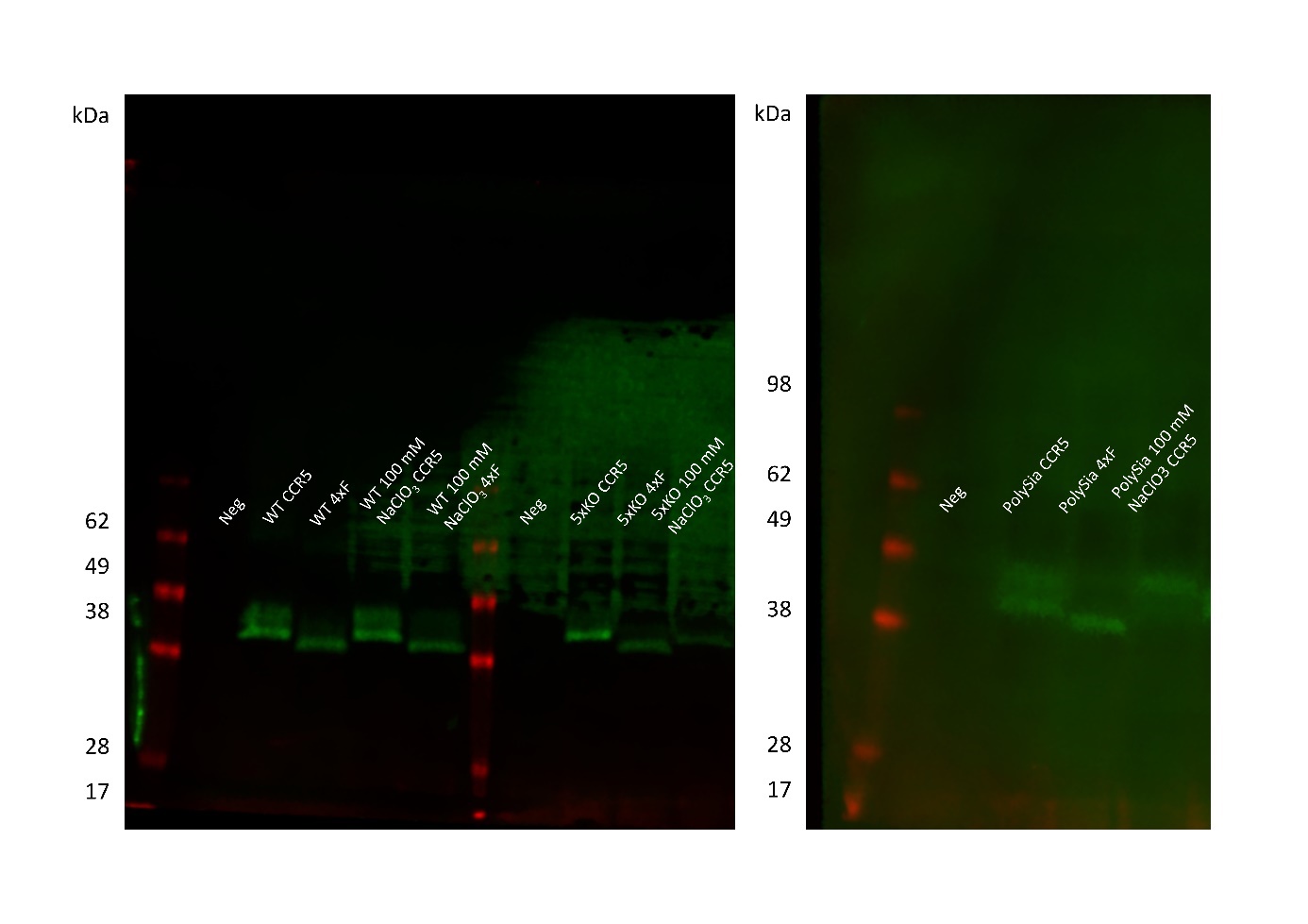


**Full western blots of figure 7**

A western blot was performed to verify whether any difference can be observed in glycosylation status with the two methods of removal of O-glycosylation. CHO cells were transfected with FLAG-tagged CCR5, 4xF CCR5 or CCR5 and treated with 100 mM NaClO_3_, harvested and analyzed by western blot. It was revealed that the 4xF mutant in all cell lines apparently not only removes tyrosine sulfation, but also O-glycosylation. The cells treated with 100 mM NaClO_3_ still have O-glycosylation. A down shift was detected in the cell lines transfected with the 4xF sequence. This is likely due to alterations in SDS binding which has been reported before in studies with tyrosine to phenylalanine mutations in GPCRs (13).

**Table S5**

| **Chemokine receptor** | **Chemokine** | **Cell line** | **EC50 (nM)** | | **95% confidence interval of the EC50 (nM)** |
| --- | --- | --- | --- | --- | --- |
| CCR5 | CCL3 | WT | | 0.91 | [0.69, 1.18] |
|  |  | 3XKO | | 0.71 | [0.47, 1.06] |
|  |  | T1KO | | 0.82 | [0.57, 1.17] |
|  |  | T2KO | | 0.56 | [0.46, 0.67] |
|  |  | T11KO | | 1.24 | [0.91, 1.70] |
|  | CCL5 | WT | | 1.50 | [0.94, 2.44] |
|  |  | 3XKO | | 1.47 | [1.05, 2.10] |
|  |  | T1KO | | 1.06 | [0.72, 1.59] |
|  |  | T2KO | | 2.06 | [1.49, 2.88] |
|  |  | T11KO | | 2.33 | [1.43, 3.90] |
|  | CCL8 | WT | | 2.39 | [1.90, 3.04] |
|  |  | 3XKO | | 1.41 | [0.74, 2.80] |
|  |  | T1KO | | 1.95 | [0.89, 4.66] |
|  |  | T2KO | | 1.90 | [1.16, 3.21] |
|  |  | T11KO | | 3.46 | [1.38, 8.62] |

**EC50 of the cells with single GALNT KOs with O-glycosylation**

The 95% confidence interval of the EC50 values based on the concentration-response curves of the CCR5 Gα_i_-signaling induced by CCL3, CCL5 or CCL8 in the different CHO cell lines (WT, 3XKO, 5XKO, T1KO, T2KO or T11KO).

| **Chemokine** | **Cell lines compared** | | **P-value** | |
| --- | --- | --- | --- | --- |
| CCL3 | WT | 3XKO | <0.0001 | **** |
|  |  | T1KO | <0.0001 | **** |
|  |  | T11KO | 0.0002 | *** |
|  | 3XKO | T2KO | <0.0001 | **** |
|  |  | T11KO | 0.0004 | *** |
|  | T2KO | T1KO | <0.0001 | **** |
|  |  | T11KO | <0.0001 | **** |
| CCL5 | WT | 3XKO | 0.0096 | ** |
|  |  | T1KO | <0.0001 | **** |
|  |  | T11KO | 0.0106 | * |
|  | T2KO | 3XKO | 0.0177 | * |
|  |  | T1KO | <0.0001 | **** |
|  |  | T11KO | 0.0193 | * |
| CCL8 | WT | T1KO | 0.0026 | ** |
|  | T1KO | T2KO | 0.0010 | *** |

**Table S6**

**Statistical analysis for Gαi-signaling measured by BRET after removal of tyrosine sulfation**

Statistical analysis was performed to determine significant differences in CCR5-induced Gα_i_-signaling after activation by 100 nM CCL3, CCL5 or CCL8 between the cell lines. A two-way ANOVA test was performed, followed by a multiple-comparison Tukey test for the main column effects by computing the means of each data set column. P-values are only listed for the cell lines that showed statistically significant differences. The significance level is indicated by stars next to the p-value; ****p-value<0.0001, ***p-value<0.001, **p-value<0.01 and *p-value≤0.05.

**Fig. S5**


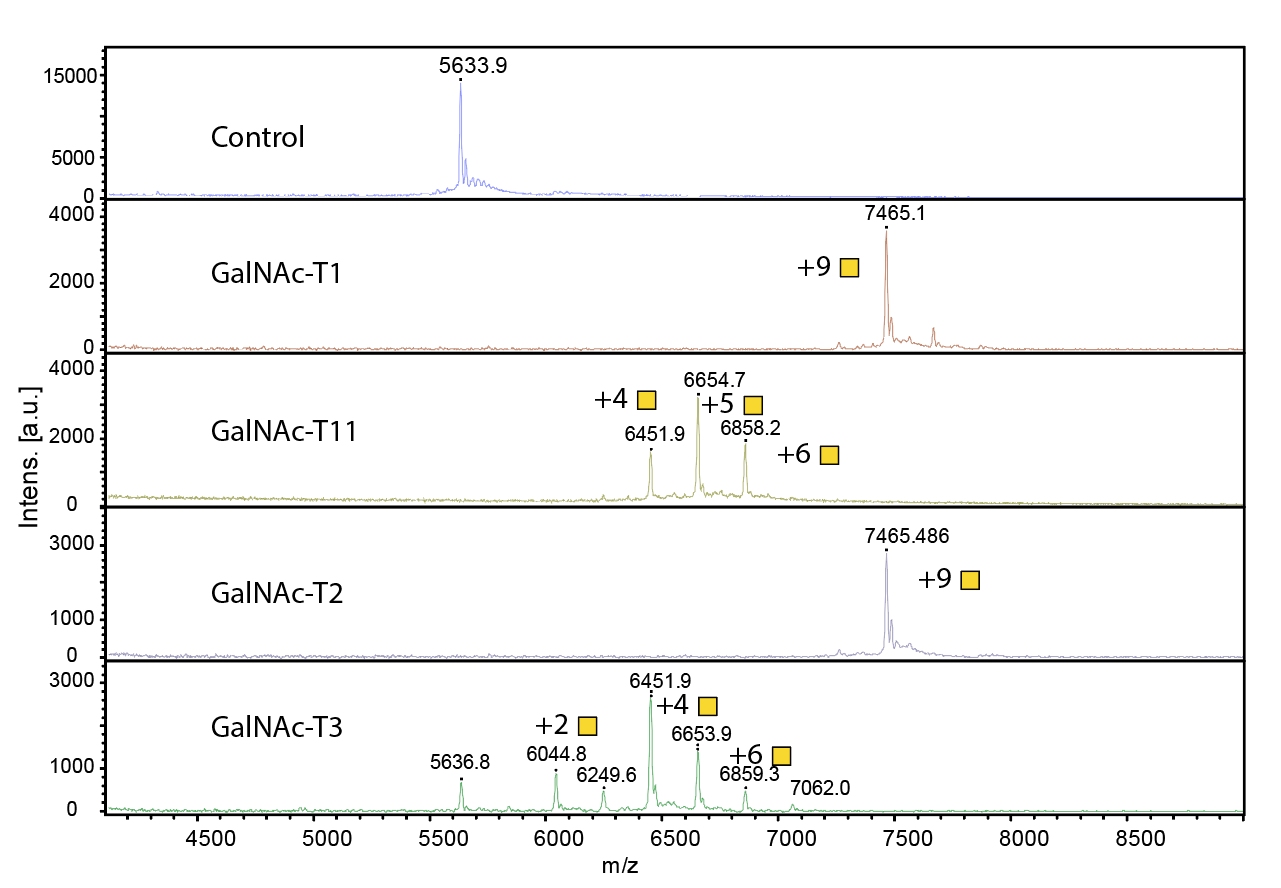


**MALDI-TOF analysis of in vitro glycosylation reactions of GalNAc-T1, -T2, -T3 and T11 with a MUC1-60 mer peptide**

The MUC1-60 mer peptide were incubated overnight with or without recombinant GalNAc-Ts as indicated next to each spectrum. After incubation, reactions were analyzed by MALDI-TOF and revealed that all enzymes are active as they could glycosylate the peptide at multiple positions. The numbers of incorporated GalNAcs are shown next to respective peaks.
